# Association between the ratio of triglyceride to high-density lipoprotein cholesterol (TG/HDL-c) and chronic kidney disease risk: a large-scale cross-sectional study in a Chinese population

**DOI:** 10.3389/fmed.2025.1629303

**Published:** 2025-10-31

**Authors:** Yuheng Liao, Qijun Wan, Haofei Hu, Haiying Song

**Affiliations:** ^1^Department of Nephrology, The First Affiliated Hospital of Shenzhen University, Shenzhen, Guangdong, China; ^2^School of Medicine, Shenzhen University, Shenzhen, Guangdong, China; ^3^Department of Nephrology, Shenzhen Second People’s Hospital, Shenzhen, Guangdong, China

**Keywords:** Chronic kidney disease, triglyceride/high-density lipoprotein ratio, cross-sectional study, non-linear association, sensitivity analysis

## Abstract

**Objective:**

The association between lipid metabolism markers, particularly the TG/HDL-c ratio, and kidney dysfunction has not been thoroughly investigated in Chinese wellness examination cohorts. This study examines the correlation between these parameters and evaluates the role of TG/HDL-c as an independent predictor of CKD.

**Methods:**

Our multicenter investigation encompassed data from 33,850 consecutive participants across eight major Chinese metropolitan areas (Shanghai, Guangzhou, Wuhan, Dalian, Zhengzhou, Lanzhou, Luzhou, and Guangxi). To evaluate the relationship between TG/HDL-c ratio and CKD, we employed multiple statistical approaches. The primary analysis utilized binary logistic regression to assess the independent association between these variables. We further characterized the relationship pattern through generalized additive modeling (GAM) with smooth curve fitting, implementing the penalty spline method. The reliability of our findings was validated through comprehensive sensitivity analyses, complemented by stratified subgroup evaluations.

**Results:**

The average age of the study participants was (57.65 ± 9.28) years, with females accounting for 67.15%. The prevalence of CKD was 15.9%, and the median TG/HDL-C ratio was 1.06 (0.69–1.65). After adjusting for covariates, the results indicated a positive correlation between the TG/HDL-C ratio and CKD (OR = 1.17, 95% CI: 1.13, 1.21). There was also a nonlinear relationship between the TG/HDL-C ratio and CKD, with an inflection point at 1.086. The effect sizes on either side of the inflection point were 1.800 (1.542, 2.100) and 1.049 (1.049, 1.411), respectively. Sensitivity analysis confirmed the robustness of the study findings.

**Conclusion:**

This study demonstrates a positive and non-linear relationship between TG/HDL-c ratio and CKD in the Chinese health check-up population. TG/HDL-c ratio is strongly related to CKD when TG/HDL-c ratio is more than 1.086. It makes sense to reduce the TG/HDL-c ratio level below the inflection point from a treatment perspective.

## 1 Background

Chronic kidney disease (CKD) has emerged as a significant public health challenge, affecting 13.4% (11.7–15.1%) of the global population. Current epidemiological data indicate that between 49.02 and 70.83 million individuals are at risk of progressing to End-Stage Renal Disease (ESRD) (1). Currently, China has the highest number of CKD patients in the world, approximately 119.5 million (2). Despite access to dialysis interventions, CKD patients continue to experience substantial health challenges, including elevated cardiovascular disease (CVD) risk, compromised life quality, and significant mortality rates approximating 50% (3). Therefore, early screening and management of risk factors associated with CKD are crucial for disease prevention.

Extensive research has established the pivotal influence of lipid profiles on CKD initiation and progression (4, 5), with dyslipidemia manifesting in approximately 82% of affected individuals (6). Among various lipid parameters, the triglyceride to high-density lipoprotein cholesterol (TG/HDL-c) ratio has emerged as a significant metabolic marker. Clinical evidence supports its predictive utility for multiple outcomes, including diabetes (HR: 1.85, 95% CI: 1.46–2.35) (7), chronic kidney disease (OR: 1.54, 95% CI: 1.21–1.96) (8), adverse renal events (9), and cardiovascular events (5, 10, 11).

However, given the multifaceted relationship between lipid metabolism and renal function, individual lipid parameters may insufficiently characterize the metabolic dysregulation in CKD (6). Recent investigations have established that elevated TG/HDL-c ratios correlate significantly with insulin resistance (12), an established driver of CKD progression (13). Moreover, this integrated marker has shown superior diagnostic efficacy compared to traditional lipid indices in identifying early-stage renal impairment (8).

Research examining the relationship between TG/HDL-c and CKD has yielded heterogeneous results. In a notable investigation, Ji-Young Kim and colleagues (14) identified TG/HDL-c as the sole lipid-related ratio significantly associated with CKD stage ≥3 prevalence, demonstrating gender-specific associations (men: OR 1.82, 95% CI 1.09–3.03; women: OR 2.34, 95% CI 1.44–3.79; *p* < 0.05). Similarly, a prospective cohort analysis of 7,316 middle-aged and elderly Chinese participants (5) revealed an independent association between elevated TG/HDL-C ratio and declining renal function. Following multivariate adjustments, subjects in the highest TG/HDL-C tertile exhibited significantly elevated risk for reduced renal function compared to those in the lowest tertile (OR: 1.30; 95% CI: 1.03–1.65; *p* = 0.03). Conversely, contradictory evidence exists. A large-scale randomized controlled trial in 2006 demonstrated that while statin therapy effectively reduced LDL-c levels, it showed no significant impact on CKD progression (15). Further research (16) suggested that dyslipidemia may not constitute an independent risk factor for long-term outcomes in patients with CKD stages 3–4. Moreover, the generalizability of existing evidence remains limited, as previous investigations predominantly focused on specific populations, including middle-aged and older adults, individuals with cardiovascular conditions, hypertension, or diabetes (17–19).

Furthermore, current evidence presents conflicting patterns in the TG/HDL-c ratio and CKD relationship, with investigations documenting both linear associations (5, 20) and more intricate non-linear correlations (21). This highlights the need for further investigation to better understand the association between TG/HDL-c ratio and CKD. Additionally, previous studies predominantly defined CKD solely based on eGFR <60 mL/min/1.73 m^2^, without considering urinary albumin-to-creatinine ratio (UACR). This single-criterion approach might have underestimated the true prevalence of CKD (17, 22, 23). In the present study, we adopted a more comprehensive definition of CKD according to the latest KDIGO guidelines (24), incorporating both eGFR <60 mL/min/1.73 m^2^ and UACR ≥30 mg/g criteria. This dual-criterion approach provides a more accurate assessment of kidney dysfunction, as it captures both structural and functional abnormalities of the kidney.

To address current research limitations, this investigation evaluates the relationship between TG/HDL-c ratio and CKD utilizing data from a comprehensive multi-center health examination cohort representative of the Chinese population. Our hypothesis posits a significant association between these parameters, potentially characterized by non-linear patterns not previously elucidated. Through examination of this demographically diverse cohort, this study aims to enhance the clinical utility of TG/HDL-c ratio as a CKD risk marker and facilitate the development of refined risk assessment strategies.

## 2 Methods

### 2.1 Study design

The study employed a cross-sectional design to evaluate the association between TG/HDL-c ratio (designated as the primary predictor) and CKD status (outcome variable, categorized dichotomously: CKD = 1, non-CKD = 0).

### 2.2 Data source

We downloaded the raw data freely from,^1^ provided by Ye et al. (25). Under the terms of the Creative Commons Attribution License, which allows unrestricted use, distribution, and reproduction in any medium, provided the original author and source are credited (25).

### 2.3 Study population

To reduce selection bias, the REACTION study enrolled 33,850 consecutive participants across eight Chinese metropolitan centers (Dalian, Guangzhou, Zhengzhou, Lanzhou, Luzhou, Wuhan, Guangxi, and Shanghai). Standardized questionnaires were administered to collect comprehensive baseline data, including medical history, physical activity patterns, and lifestyle behaviors (smoking and alcohol consumption). The study protocol received approval from the Human Research Committee of Ruijin Hospital, Shanghai Jiao Tong University School of Medicine. All participants provided written informed consent before study initiation (25).

The initial study (25) employed the following criteria for exclusion:(1) Individuals with primary kidney disease; (2) Participants on daily ACEI or ARB medications; (3) Those with a fallacious self-reported sleep duration (<4 h or >12 h). Ultimately, the initial research involved 33,850 individuals in the analysis.

**Figure 1:**
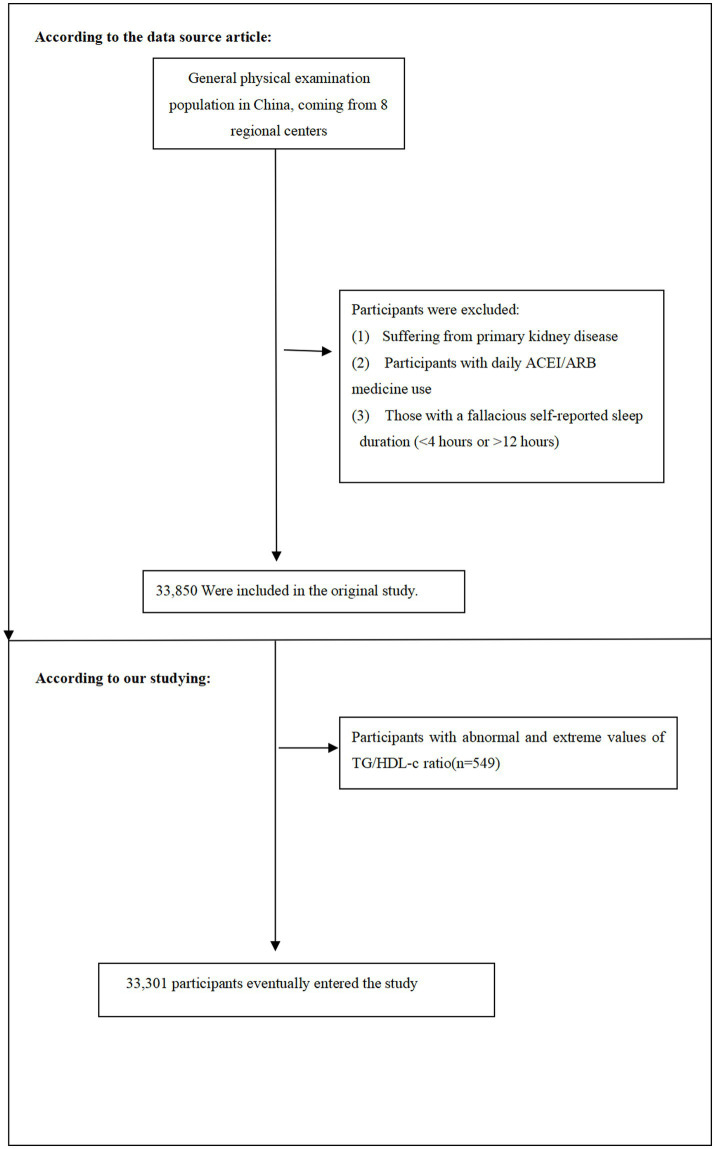
Flow chart of study participant selection.

In this current study, following the original research, we removed individuals with TG/HDL-c values beyond three standard deviations from the mean (*n* = 549) (21). 33,301 people in total participated in the present study at the end. Figure 1 shows illustrate the process of selecting participants.

The flow chart illustrates the selection process of study participants from the REACTION study. Initially, 33,850 participants were recruited. After excluding participants with TyG-BMI values beyond three standard deviations from the mean (*n* = 549), 33,301 participants were included in the final analysis.

### 2.4 Variables

#### 2.4.1 Data collection and measurements

Demographic characteristics, medical history, and lifestyle parameters (physical activity, smoking status, and alcohol consumption) were assessed using a standardized questionnaire. Smoking and alcohol consumption were stratified into three categories: regular, occasional, or never users. Missing data were identified for lifestyle variables, including smoking (*n* = 275), alcohol consumption (*n* = 262), and physical activity (*n* = 127). To optimize statistical power and mitigate potential selection bias, participants with missing data were retained and classified as “Not recorded” rather than excluded from analyses (26).

Baseline assessments were conducted by trained healthcare professionals following standardized protocols. Anthropometric measurements comprised height (stadiometer; precision: 0.1 cm), weight (calibrated electronic scales; precision: 0.1 kg), and waist circumference (measured at the horizontal plane between the anterior superior iliac spine and inferior costal margin). Blood pressure measurements were obtained in triplicate at 1-min intervals following a 5-min seated rest period, with the arithmetic mean utilized for analyses. Mean arterial pressure (MAP) was calculated from the triplicate measurements. Obesity was defined according to established criteria (BMI ≥ 28 kg/m2) (27). Hypertension was classified based on systolic blood pressure ≥130 mmHg, diastolic blood pressure ≥80 mmHg, or documented hypertension history.

Following an 8-h overnight fast, morning blood samples were collected at baseline and 120 min post-challenge. Biochemical parameters assessed included triglycerides (TG), total cholesterol (TC), high-density lipoprotein cholesterol (HDL-c), low-density lipoprotein cholesterol (LDL-c), serum creatinine (Scr), alanine aminotransferase (ALT), aspartate aminotransferase (AST), and *γ*-Glutamyl transpeptidase (GGT). Diabetes mellitus (DM) was defined according to established criteria (28): fasting plasma glucose (FPG) ≥ 7.0 mmol/L, 2-h postprandial blood glucose (PBG) ≥ 11.1 mmol/L, or documented diabetes history.

#### 2.4.2 Calculation and assessment of TG/HDL-c ratio

At baseline, we collected and recorded the TG/HDL-c ratio as a continuous variable. The calculation method for the TG/HDL-c ratio was performed according to established protocols (14), where serum triglyceride concentration (measured in mmol/L) was divided by high-density lipoprotein cholesterol level (measured in mmol/L).

#### 2.4.3 Definition of chronic kidney disease

CKD was defined according to established diagnostic criteria as either the presence of albuminuria (urinary albumin-to-creatinine ratio [UACR] ≥ 30 mg/g) or reduced estimated glomerular filtration rate (eGFR <60 mL/min/1.73 m^2^) (29). For UACR assessment, morning urine samples were collected. The estimation of glomerular filtration rate was performed using the Modification of Diet in Renal Disease (MDRD) equation30: eGFR (ml/min*1.73 m^2^) = 186*Scr (mg/dl)^-1.154^*Age (years)^-0.203^(female*0.742) (30).

### 2.5 Assessment of covariates

Variable selection in this study was based on preliminary investigation, clinical expertise, and previous research examining CKD risk factors (21, 31, 32). The analyzed covariates were categorized into two groups: (1) Continuous variables: age, WC, LDL-c, TC, AST, ALT, GGT, HDL-c. (2) Categorical variables: demographic factors (gender), lifestyle characteristics (smoking and drinking habits), chronic disease history (including hypertension, diabetes mellitus, and malignancy).

### 2.6 Statistical analysis

Study participants were stratified into quartiles according to their TG/HDL-c ratio values. In descriptive statistical analyses, continuous variables with normal distribution were presented as mean ± standard deviation, while those with skewed distributions were expressed as median (range). For categorical variables, frequencies and percentages were reported. Statistical comparisons across TG/HDL-c ratio quartiles were conducted using One-Way ANOVA for normally distributed variables, Kruskal-Wallis H tests for variables with skewed distributions, and χ2 tests for categorical variables.

The assessment of potential covariate collinearity was performed using variance inflation factors (VIF) (33). VIF values were calculated according to the formula: 1/(1-R^2^), where R^2^ was obtained from sequential linear regression models in which each variable was treated as the dependent variable against all remaining variables. To address collinearity concerns, variables demonstrating VIF > 5 were excluded from subsequent multiple regression analyses (detailed results presented in Supplementary Table S1).

The association between TG/HDL-c ratio and CKD was investigated using univariate and multivariate binary logistic regression analyses, following the STROBE statement guidelines (34, 35). The analyses included an unadjusted model without covariate adjustment, a minimally adjusted model accounting for sociodemographic variables (age, gender, and BMI), and a fully adjusted model incorporating all covariates listed in Table 1 (gender, age, BMI, LDL, ALT, AST, GGT, smoking and drinking status, hypertension, and cancer history). The strength of associations was quantified using odds ratios (OR) with corresponding 95% confidence intervals (CI).

**Table 1:** The characteristics of participants.

TG/HDL-c quartile	Q1(<0.69)	Q2(0.69–1.06)	Q3(1.06–1.65)	Q4(> = 1.65)	*P*-value
Participants	8,324	8,326	8,324	8,327	
Age (years)	56.16 ± 9.33	57.70 ± 9.29	58.33 ± 9.27	58.41 ± 9.05	<0.001
Gender, *n* (%)					<0.001
Male	2,129 (25.58)	2,546 (30.58)	2,881 (34.61)	3,384 (40.64)	
Female	6,195 (74.42)	5,780 (69.42)	5,443 (65.39)	4,943 (59.36)	
BMI (kg/m^2^)	23.21 ± 3.52	24.29 ± 3.56	25.19 ± 3.60	25.86 ± 3.54	<0.001
MAP (mmHg)	92.55 ± 12.57	94.92 ± 12.67	96.61 ± 12.51	98.41 ± 12.46	<0.001
WC (cm)	81.93 ± 9.90	85.10 ± 9.66	87.76 ± 9.62	89.73 ± 9.17	<0.001
HC (cm)	94.46 ± 7.71	96.40 ± 7.63	98.09 ± 7.72	98.99 ± 7.52	<0.001
HDL-c (mmol/L)	1.63 ± 0.33	1.37 ± 0.26	1.22 ± 0.23	1.07 ± 0.21	<0.001
LDL-c (mmol/L)	2.88 ± 0.83	3.08 ± 0.91	3.14 ± 0.91	2.87 ± 0.89	<0.001
TC (mmol/L)	5.01 ± 1.03	5.05 ± 1.13	5.10 ± 1.15	5.09 ± 1.17	<0.001
TG(mmol/L)	0.81 ± 0.20	1.18 ± 0.25	1.61 ± 0.34	2.69 ± 0.91	<0.001
TG/HDL-c ratio	0.51 ± 0.12	0.87 ± 0.10	1.32 ± 0.17	2.56 ± 0.87	<0.001
ALT (U/L)	15.62 ± 13.01	16.89 ± 12.04	18.39 ± 13.96	20.68 ± 14.99	<0.001
AST (U/L)	21.76 ± 11.66	22.01 ± 11.43	22.48 ± 12.32	23.21 ± 13.12	<0.001
GGT (U/L)	23.00 ± 26.31	26.79 ± 35.23	30.58 ± 37.01	37.45 ± 43.78	<0.001
Scr (mmol/L)	66.00 ± 14.18	67.42 ± 16.30	68.88 ± 15.77	71.36 ± 20.09	<0.001
eGFR (ml/min/1.73m^2^)	95.72 ± 17.91	94.59 ± 18.48	93.46 ± 18.88	92.32 ± 20.41	<0.001
UACR (mg/g)	23.10 ± 222.19	30.70 ± 430.44	34.16 ± 466.03	41.34 ± 377.12	0.022
Smoking habits, *n* (%)					<0.001
Never smoker	7,361 (89.03)	7,134 (86.43)	6,974 (84.58)	6,625 (80.22)	
Sometimes smoker	162 (1.96)	189 (2.29)	224 (2.72)	278 (3.37)	
Regular smoker	745 (9.01)	931 (11.28)	1,047 (12.70)	1,356 (16.42)	
Not record	56 (0.673)	72 (0.865)	79 (0.949)	68 (0.817)	
Drinking habits, *n* (%)					<0.001
Never drinker	6,213 (75.20)	6,142 (74.37)	6,142 (74.39)	5,967 (72.23)	
Sometimes drinker, %	1,475 (17.85)	1,581 (19.14)	1,585 (19.20)	1,650 (19.97)	
Regular drinker, %	574 (6.95)	536 (6.49)	530 (6.42)	644 (7.80)	
Not record	62 (0.745)	67 (0.805)	67 (0.805)	66 (0.793)	
Exercise habits, *n* (%)					
Yes	2018 (24.33)	1719 (20.74)	1,637 (19.72)	1,685 (20.33)	<0.001
No	6,276 (75.396)	6,568 (78.885)	6,666 (80.082)	6,605 (79.320)	
Not record	30 (0.360)	39 (0.468)	21 (0.252)	37 (0.444)	
Tumor, *n* (%)	250 (3.00)	240 (2.88)	241 (2.90)	246 (2.95)	0.965
CKD, *n* (%)	928 (11.15)	1,229 (14.76)	1,451 (17.43)	1,690 (20.30)	<0.001
Hypertension, *n* (%)	4,052 (48.68)	4,784 (57.46)	5,332 (64.06)	5,847 (70.22)	<0.001
Diabetes, *n* (%)	1,036 (12.45)	1,577 (18.94)	2074 (24.92)	2,783 (33.42)	<0.001
Obesity, *n* (%)	626 (7.52)	1,031 (12.38)	1,523 (18.30)	1839 (22.08)	<0.001

Values are *n* (%) or mean ± SD or median (quartile). BMI, body mass index; MAP, mean arterial blood pressure; WC, waist circumference; HC, hip circumference; TC, total cholesterol; TG, triglyceride; HDL-c, high-density lipoprotein cholesterol; LDL-c, low-density lipoprotein cholesterol; TG/HDL-c, ratio triglyceride/high-density lipoprotein cholesterol ratio; ALT, alanine aminotransferase; AST, aspartate aminotransferase; Scr, serum creatinine; GGT, γ-glutamyl transpeptidase; eGFR, Glomerular Filtration Rate; UACR, Random urinary protein/creatinine ratio; OR, Odds ratios; CI, confidence interval; Ref reference.

To explore potential nonlinear relationships between the TG/HDL-c ratio and CKD, generalized additive models (GAM) with penalized spline fitting were employed. When nonlinearity was detected, we utilized a recursive algorithm to identify the inflection point and conducted subsequent two-piece logistic regression analyses. Model selection was performed using the log-likelihood ratio test (36).

We conducted stratified logistic regression analyses to investigate potential effect modifications by gender, age, BMI, smoking status, drinking status, exercise habits, and cancer history. Prior to analysis, continuous variables were transformed into categorical variables using established clinical thresholds. Age was categorized into five groups (<40, 40–50, 50–60, 60–70, ≥70 years), and BMI was classified into four categories (< 18.5, ≥ 18.5 to < 24, ≥ 24 to < 28, ≥ 28 kg/m^2^). Additional categorical variables included smoking status (Regular smoker, Sometimes smoker, Never smoker), drinking status (Regular drinker, Sometimes drinker, Never drinker), exercise habits (Yes, No), and tumor history (Yes, No). Each stratified analysis incorporated adjustments for all covariates except the stratification variable itself, including gender, age, BMI, LDL-c, ALT, AST, GGT, smoking and drinking status, hypertension, and cancer history. Interaction effects were assessed through likelihood ratio tests comparing models with and without interaction terms (37, 38).

To validate our findings, we performed comprehensive sensitivity analyses. The relationship between TG/HDL-c ratio and CKD was examined using both continuous and categorical (quartiles) approaches, with trend testing to evaluate result consistency and potential non-linear associations. Considering the well-documented relationships between CKD risk and diabetes, hypertension, and obesity (39–41), we conducted additional analyses after excluding participants with these conditions. The robustness of our findings was further verified using a generalized additive model (Model IV), which incorporated the continuous covariate as a curve (42). We calculated E-values to assess the potential impact of unmeasured confounding on the association between TG/HDL-c ratio and CKD risk (43). All research was performed in accordance with relevant guidelines and regulations and in accordance with the Declaration of Helsinki. Furthermore, to address potential confounding by lipid-lowering therapy, we considered that people with high lipid levels would be susceptible to lipid-lowering therapy, we performed sensitivity analyses excluding patients who might be receiving such drugs. Specifically, we included only participants with TG < 1.7 mmol/L (44) (i.e., those with normal triglycerides who are less likely to receive lipid-lowering therapy). Similarly, we also only included participants with LDL-c < 3.12 mmol/L (45) to further determine the stability of the relationship between TG/HDL-c and the risk of CKD.

The data analysis was performed utilizing two statistical software packages, namely R (The R Foundation, http://www.R-project.org) and EmpowerStats (X&Y Solutions, Inc., Boston, MA, http://www.empowerstats.com). All statistical tests were conducted as two-sided tests, and a significance level of *p*-value < 0.05 was employed to ascertain statistical significance.

## 3 Results

### 3.1 Characteristics of participants

Table 1 provided the demographic and clinical characteristics of participants included in the study. A total of 33,301 research subjects were included in the final analysis, of which females accounted for 67.15%, with an average age of 57.65 ± 9.28 years. The median TG/HDL-C ratio was 1.06 (0.69–1.65), the average BMI was 24.64 ± 3.69 kg/m^2^. The prevalence of CKD was 15.91% (5,298/33,301). Participants were categorized into subgroups based on the quartiles of the TG/HDL-c ratio (<0.69, 0.69 to 1.06, 1.06 to 1.65, ≥1.65). There were no significant differences in tumor history among the different quartiles of the TG/HDL-c ratio (*p* value > 0.05). Compared with Q1, participants in Q4 demonstrated significantly higher levels of age, BMI, WC, HC, MAP, TC, TG, ALT, AST, GGT, UACR, and TG/HDL-c ratio, with greater proportions of males, hypertension, diabetes and tobacco/alcohol users (Table 1).

**Figure 2:**
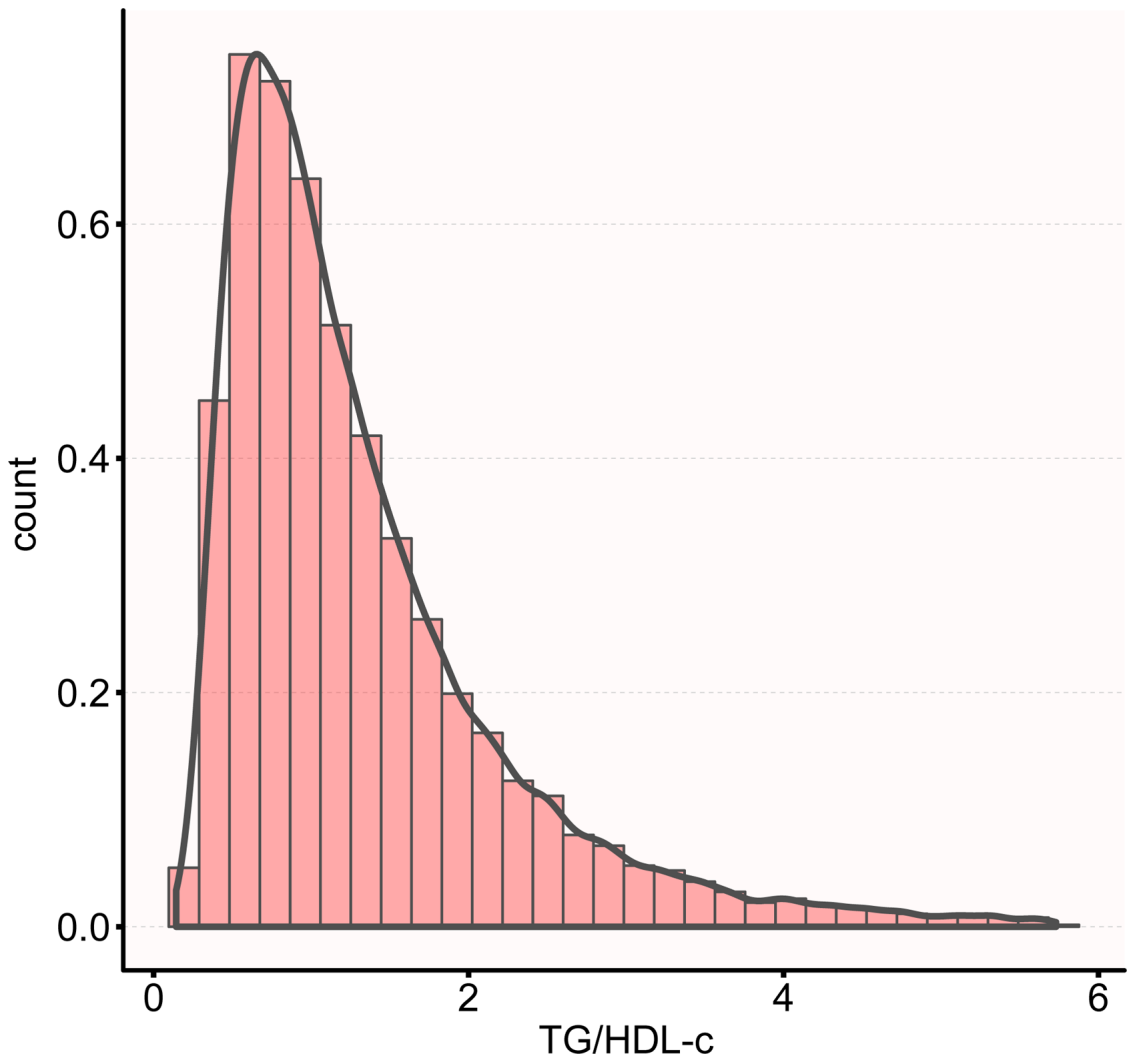
Distribution of TG/HDL-c ratio in the study population.

**Figure 3:**
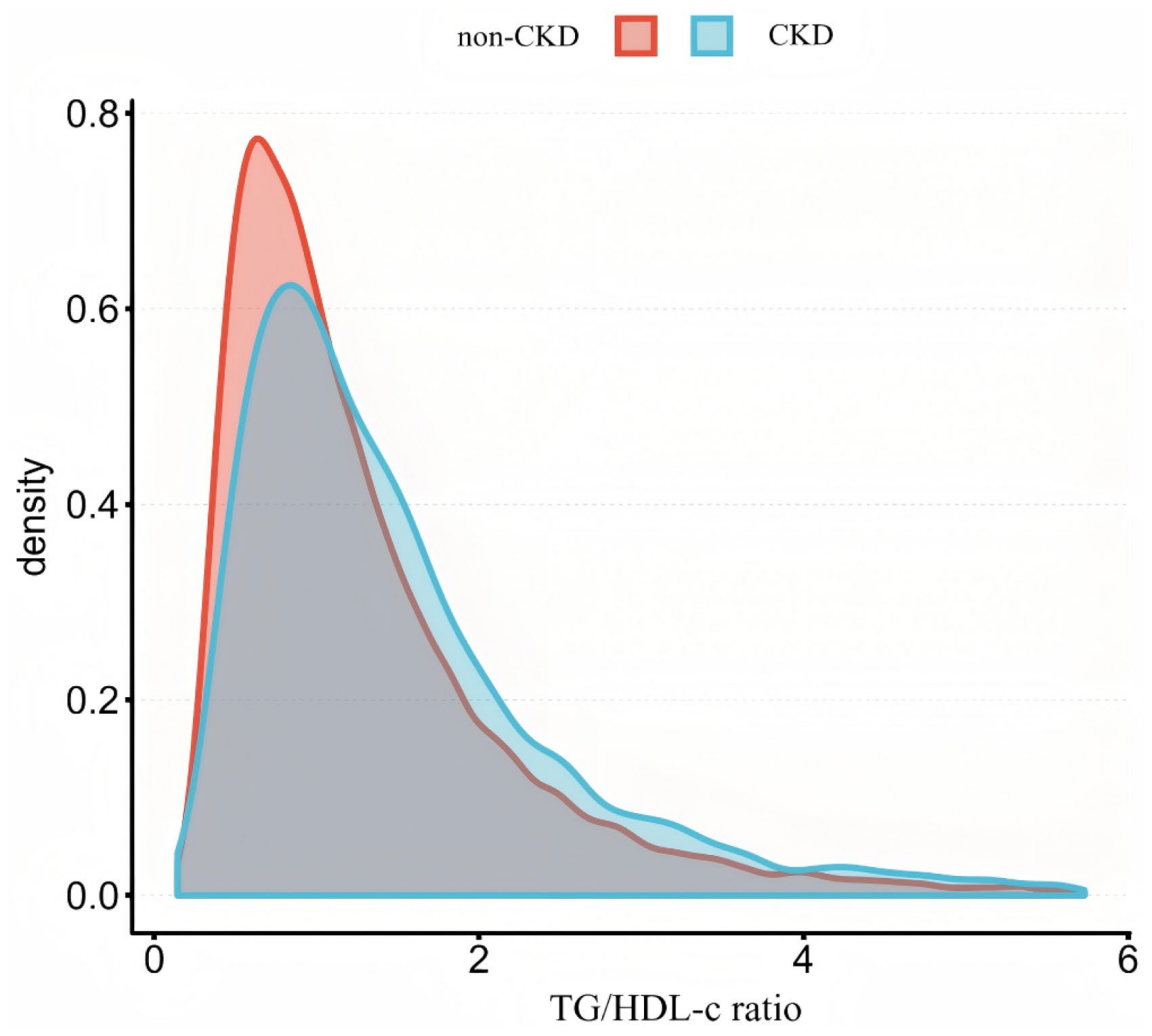
Comparison of TG/HDL-c ratio distribution between CKD and non-CKD groups.

Figure 2 shows the distribution of TG/HDL-c ratio levels, which is right-skewed distribution, ranging from 0.143 to 5.730, with median of 1.314. Participants were classified into CKD and non-CKD groups, with TG/HDL-c ratio values compared between groups (Figure 3). The results showed that TG/HDL-c ratio levels were significantly higher in the CKD group.

The histogram shows the distribution of TG/HDL-c values among all participants. TG/HDL-c ratio exhibited a Right-skewed distribution with values ranging from 0.143 to 5.730, with a mean value of 1.314.

Figure 3 illustrates the comparison of the distribution of TG/HDL-c ratio between participants with and without CKD. The results indicated that the CKD group demonstrated higher TG/HDL-c ratio levels compared to the non-CKD group.

### 3.2 The prevalence rate of CKD

**Figure 4:**
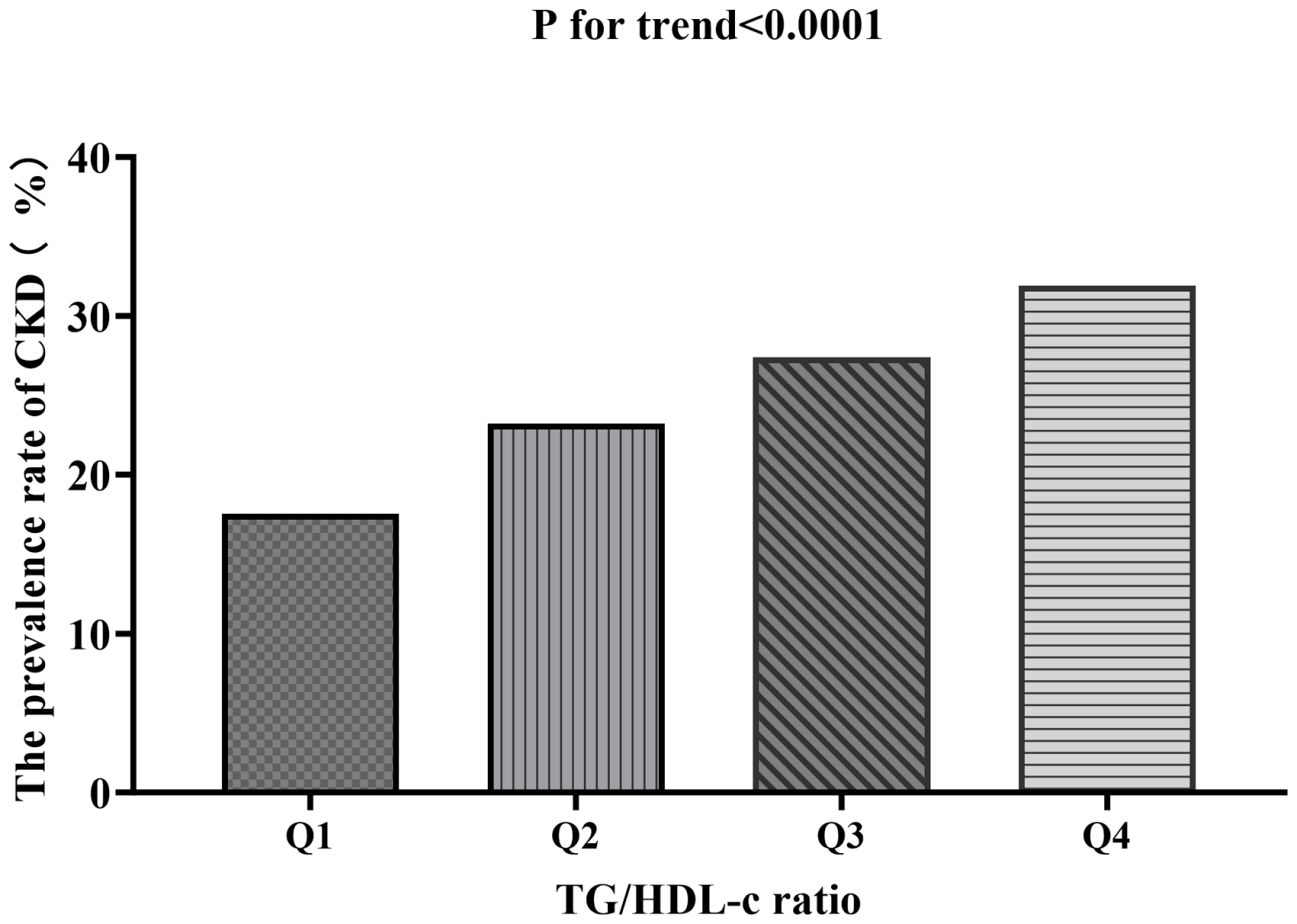
Prevalence of CKD across TG/HDL-c ratio quartiles.

Table 2 shows that among all participants, a total of 5,298 individuals had chronic kidney disease (CKD). The overall prevalence rate was 15.9% (15.5–16.3%). The prevalence of CKD varied across the four groups based on the TG/HDL-c ratio, with a significant increase in incidence compared to the Q1 (<0.69) group. Specifically, the prevalence rates for each TG/HDL-c ratio group were as follows: 15.9% (15.5–16.3%), 11.1% (10.4–11.8%), 14.8% (14.0–15.5%), 17.4% (16.6–18.2%), and 20.3% (19.4–21.2%). Notably, participants in the high TG/HDL-c ratio group had a higher prevalence of CKD compared to those in the lowest TG/HDL-c ratio group (*p* < 0.0001 for trend; Figure 4).

**Table 2:** Prevalence rate of CKD.

TG/HDL-c ratio	Participants (n)	CKD events (n)	Cumulative incidence rate (95% CI) (%)
Total	33,301	5,298	15.9 (15.5–16.3)
Q1	8,324	928	11.1 (10.4–11.8)
Q2	8,326	1,299	14.8 (14.0–15.5)
Q3	8,324	1,451	17.4 (16.6–18.2)
Q4	8,327	1,690	20.3 (19.4–21.2)
P for trend	<0.0001		

Bar chart showing the prevalence of CKD across different TG/HDL-c ratio quartiles. The prevalence rates were 17.52, 23.20, 27.39, and 31.9% from the lowest to highest quartile, demonstrating a significant increasing trend (p < 0.0001). Compared with the lowest TG/HDL-c ratio group, participants with a high TG/HDL-c ratio had a higher cumulative prevalence of CKD (*p* < 0.001 for trend).

**Figure 5:**
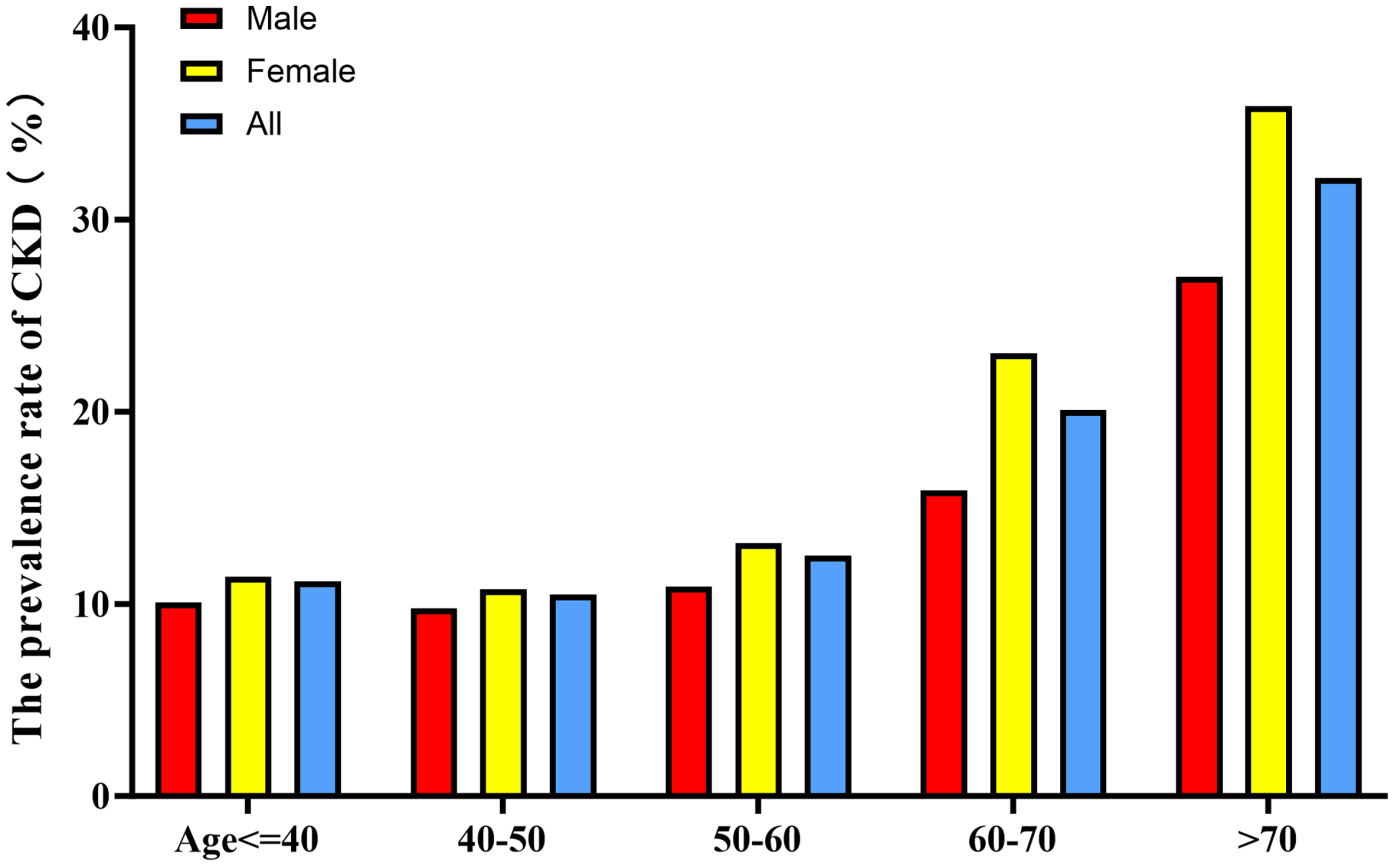
Age- and sex-specific prevalence of CKD.

When stratified by 10-year age intervals, the prevalence of CKD was consistently higher in the female population compared to their male counterparts across all age groups (Figure 5). Furthermore, a significant age-associated increase in CKD risk was observed in both male and female participants.

Bar graph showing CKD prevalence stratified by age groups (10-year intervals) and sex. Female participants showed higher CKD prevalence than males across all age groups. The prevalence increased with age in both sexes, with a more pronounced trend in females.

The results of univariate analyses using a binary logistic regression model.

In univariate analysis, HDL-c, LDL-c, eGFR, regular smoking, and regular drinking showed negative associations with CKD risk. In contrast, age, gender, BMI, WC, HC, MAP, TG, TG/HDL-c ratio, ALT, AST, GGT, Scr, UACR, hypertension, diabetes, obesity, never smoking, and never drinking were positively associated with CKD risk. No significant associations were observed between CKD risk and tumor history, TC, or regular smoking status (all *p* < 0.0001, detailed results shown in Table 3).

**Table 3:** The results of univariate analysis.

Univariate analysis of risk factors associated with clinical outcomes	Statistics	OR (95% CI)	*P* value
Age (years)	57.652 ± 9.279	1.050 (1.047, 1.053)	<0.00001
Gender, *n* (%)			
Male	10,940 (32.852)		Ref
Female	22,361 (67.148)	1.194 (1.120, 1.273)	<0.00001
BMI (kg/m^2^)	24.637 ± 3.692	1.036 (1.028, 1.044)	<0.00001
WC (cm)	86.131 ± 10.027	1.018 (1.015, 1.021)	<0.00001
HC(cm)	96.982 ± 7.839	1.016 (1.013, 1.020)	<0.00001
MAP (mmHg)	95.623 ± 12.737	1.027 (1.024, 1.029)	<0.00001
HDL-c (mmol/L)	1.323 ± 0.335	0.666 (0.609, 0.730)	<0.00001
LDL-c (mmol/L)	2.993 ± 0.893	0.949 (0.918, 0.981)	0.00200
TC (mmol/L)	5.062 ± 1.123	0.991 (0.965, 1.017)	0.48412
TG (mmol/L)	1.575 ± 0.872	1.290 (1.251, 1.330)	<0.00001
TG/HDL-c ratio	1.315 ± 0.897	1.266 (1.230, 1.304)	<0.00001
ALT (U/L)	17.896 ± 13.676	1.004 (1.002, 1.006)	0.00009
AST (U/L)	22.367 ± 12.163	1.008 (1.006, 1.010)	<0.00001
GGT (U/L)	29.459 ± 36.517	1.002 (1.001, 1.003)	<0.00001
Scr (mmol/L)	68.417 ± 16.842	1.031 (1.029, 1.033)	<0.00001
eGFR(ml/min/1.73m^2^)	94.023 ± 18.984	0.971 (0.969, 0.972)	<0.00001
UACR, mg/g	32.326 ± 385.412	1.272 (1.263, 1.281)	<0.00001
Hypertension, *n* (%)			
Yes	20,015 (60.103)	1.956 (1.833, 2.087)	<0.00001
No	13,286 (39.897)		Ref
Diabetes, *n* (%)			
Yes	7,470 (22.432)	2.153 (2.020, 2.294)	<0.00001
No	25,831 (77.568)		Ref
Obesity, *n* (%)			
BMI > = 28 kg/m^2^	5,019 (15.072)	1.361 (1.261, 1.470)	<0.00001
BMI < 28 kg/m^2^	28,282 (84.928)		Ref
Tumor, *n* (%)			
Yes	977 (2.934)		Ref
No	32,324 (97.066)	1.011 (0.849, 1.204)	0.89861
Smoking habits, *n* (%)			
Never smoker	28,094 (85.066)		Ref
Sometimes smoker	853 (2.583)	0.958 (0.795, 1.156)	0.65576
Regular smoker	4,079 (12.351)	0.810 (0.736, 0.890)	0.00001
Not record	275 (0.8)	0.750 (0.525, 1.071)	0.11309
Drinking habits, *n* (%)			
Never drinker	24,464 (74.046)		Ref
Sometimes drinker	6,291 (19.041)	0.705 (0.650, 0.765)	<0.00001
Regular drinker	2,284 (6.913)	0.725 (0.638, 0.822)	<0.00001
Not record	262 (0.787)	0.775 (0.544, 1.104)	0.15781
Exercise habits, *n* (%)			
Yes	7,059 (21.279)		Ref
No	26,115 (78.721)	1.724 (1.589, 1.871)	<0.00001
Not record	127 (0.381)	1.729 (1.085, 2.754)	0.02120

Values are *n* (%) or mean ± SD or median (quartile). BMI, body mass index; MAP, mean arterial blood pressure; WC, waist circumference; HC, hip circumference; TC, total cholesterol; TG, triglyceride; HDL-c, high-density lipoprotein cholesterol; LDL-c, low-density lipoprotein cholesterol; TG/HDL-c ratio, triglyceride/high-density lipoprotein cholesterol ratio; ALT, alanine aminotransferase; AST, aspartate aminotransferase; Scr, serum creatinine; GGT, γ-glutamyl transpeptidase; eGFR, Glomerular Filtration Rate; UACR, Random urinary protein/creatinine ratio; OR, Odds ratios, CI, confidence interval, Ref, reference.

The results of multivariate analyses using the binary logistic regression model.

The relationship between TG/HDL-c ratio and CKD risk was examined using three binary logistic regression models. In the unadjusted model (Model I), each unit increase in TG/HDL-c ratio was associated with a 26% higher risk of CKD (OR = 1.26, 95% CI 1.22 to 1.30). The minimally adjusted model (Model II), which accounted for demographic variables, showed that each unit increase in TG/HDL-c ratio was associated with a 25% increased risk of CKD (OR = 1.25, 95% CI 1.21 to 1.29). In the fully adjusted model (Model III), each unit increase in TG/HDL-c ratio corresponded to a 17% higher risk of CKD (OR = 1.17, 95% CI 1.13 to 1.21). The narrow confidence intervals across all models suggest robust associations between TG/HDL-c ratio and CKD risk (Table 4).

**Table 4:** Relationship between TG/HDL-c ratio and CKD under different models.

Variable	Model I (OR, 95%CI, P)	Model II(OR, 95%CI, P)	Model III (OR, 95%CI, P)	Model IV (OR, 95%CI, P)
TG/HDL-c	1.2665 (1.2296, 1.3044) < 0.00001	1.2554 (1.2166, 1.2955) < 0.00001	1.1754 (1.1371, 1.2151) < 0.00001	1.1890 (1.1497, 1.2298) < 0.00001
TG/HDL-c (Quintile)				
Q1	Ref	Ref	Ref	Ref
Q2	1.3801 (1.2598, 1.5120) < 0.00001	1.2857 (1.1715, 1.4110) < 0.00001	1.2732 (1.1583, 1.3994) < 0.00001	1.3063 (1.1877, 1.4366) < 0.00001
Q3	1.6826 (1.5398, 1.8386) < 0.00001	1.5230 (1.3897, 1.6691) < 0.00001	1.4493 (1.3193, 1.5922) < 0.00001	1.4963 (1.3608, 1.6454) < 0.00001
Q4	2.0294 (1.8609, 2.2131) < 0.00001	1.8610 (1.6995, 2.0379) < 0.00001	1.6232 (1.4771, 1.7837) < 0.00001	1.6938 (1.5390, 1.8643) < 0.00001
P for trend	<0.00001	<0.00001	<0.00001	<0.00001

Model I: we did not adjust other covariates. Model II: we adjust age; sex; BMI. Model III: we adjust age; sex; BMI; LDL; ALT; AST; WC; GGT; smoking habits, drinking habits; diabetes, hypertension, tumor. Model IV: we adjust age(Smooth); sex; BMI(Smooth); LDL-c(Smooth); ALT(Smooth); AST(Smooth); WC(Smooth); GGT(Smooth);smoking habits, drinking habits; diabetes, hypertension, tumor. OR, Odds ratios; CI, confidence; Ref, reference.

### 3.3 Sensitivity analysis

To confirm the robustness of our findings, we conducted a series of sensitivity analyses by transforming TG/HDL-c ratio into a categorical variable based on quartiles and incorporating it into the model. In these analyses, we examined CKD risk across TG/HDL-c ratio quartiles (Table 4). In the unadjusted model (Model I), compared with the lowest quartile, the ORs for CKD were 1.3801 (95% CI, 1.2598–1.5120), 1.6826 (95% CI, 1.5398–1.8368), and 2.0294 (95% CI, 1.8609–2.2131) for the second, third, and fourth quartiles, respectively. These associations persisted after adjusting for age, sex, BMI, LDL-c, ALT, AST, WC, GGT, smoking, drinking, diabetes, hypertension, and tumor history (Model II). In the fully adjusted model (Model III), the ORs were 1.2732 (95% CI, 1.1583–1.3994), 1.4493 (95% CI, 1.3193–1.5922), and 1.6232 (95% CI, 1.4771–1.7837) for the second, third, and fourth quartiles, respectively (P for trend <0.00001). The non-uniform effect size trends across quartiles suggested a potential non-linear relationship between TG/HDL-c ratio and CKD risk.

Further analyses were conducted using a generalized additive model (GAM) to incorporate the continuous covariate as a smoothing curve. The results from Model IV remained consistent with those from the fully adjusted model (Model III) (OR = 1.1890; 95% CI, 1.1497–1.2298; *p* < 0.00001) (Table 4). To assess the robustness to unmeasured confounding, we calculated the E-value, which was 1.68. This value suggests that the observed association between TG/HDL-c ratio and CKD is relatively robust to potential unmeasured confounding factors.

In addition, we conducted several supplementary sensitivity analyses to strengthen our research findings. When individuals with diabetes were excluded from the analysis, the results demonstrated that TG/HDL-c ratio remained significantly and positively associated with CKD risk (OR = 1.1848, 95% CI: 1.1230–1.2500) after adjusting for age, sex, BMI, LDL-c, ALT, AST, GGT, smoking, drinking, hypertension, and tumor history. The results were also consistent in supplemental analyses that excluded participants with hypertension or obesity (Table 5).

**Table 5:** Relationship between TG/HDL-c ratio and CKD in different sensitivity analyses.

Exposure	Adjust I (OR, 95% CI, P)	Adjust II (OR, 95% CI, P)	Adjust III (OR, 95% CI, P)
TG/HDL-c	1.1848 (1.1230, 1.2500) < 0.000001	1.1793 (1.1346, 1.2257) < 0.000001	1.1660 (1.0810, 1.2577) 0.000070
TG/HDL-c (Quartile)			
Q1 (<0.69 mmol/L)	1.0	1.0	1.0
Q2 (0.69–1.06 mmol/L)	0.9760 (0.8008, 1.1895) 0.809704	1.1521 (1.0228, 1.2977) 0.019694	1.2638 (0.9470, 1.6868) 0.111856
Q3 (1.06–1.65 mmol/L)	1.2040 (0.9985, 1.4517) 0.051800	1.3585 (1.2104, 1.5247) < 0.000001	1.4530 (1.1086, 1.9043) 0.006784
Q4 (> = 1.65 mmol/L)	1.4142 (1.1816, 1.6927) 0.000157	1.5694 (1.4003, 1.7590) < 0.000001	1.6113 (1.2355, 2.1013) 0.000430
P for trend	<0.0001	<0.0001	<0.0001

Model I was a sensitivity analysis in participants without diabetes (*N* = 7,380). We adjust age; sex; BMI; LDL; ALT; AST; WC; GGT; smoking habits, drinking habits; hypertension and tumor. Model II was a sensitivity analysis in participants without hypertension (*N* = 1,9,794). We adjust age; sex; BMI; LDL; ALT; AST; WC; GGT; smoking habits, drinking habits; diabetes, and tumor. Model III was a sensitivity analysis in participants without obesity (N = 4,972). We adjust age; sex; BMI; LDL; ALT; AST; WC; GGT; smoking habits, drinking habits; diabetes, hypertension, tumor. OR, odds ratios; CI, confidence; Ref; reference; TG/HDL-c ratio, triglyceride/ high-density lipoprotein cholesterol ratio.

In Supplementary Table S2, we included only participants with TG < 1.7 mmol/L, and the results show that the association between TG/HDL-c and CKD remains significant (OR = 1.441, 95% CI: 1.294–1.604, *p* < 0.00001). Similarly, in Supplementary Table S3, where we only included participants with LDL-c < 3.12 mmol/L, the results also show that the association between TG/HDL-c and CKD remains significant (OR = 1.115, 95% CI: 1.072–1.160, *p* < 0.00001).

### 3.4 The nonlinearity addressed by the generalized additive model

**Figure 6:**
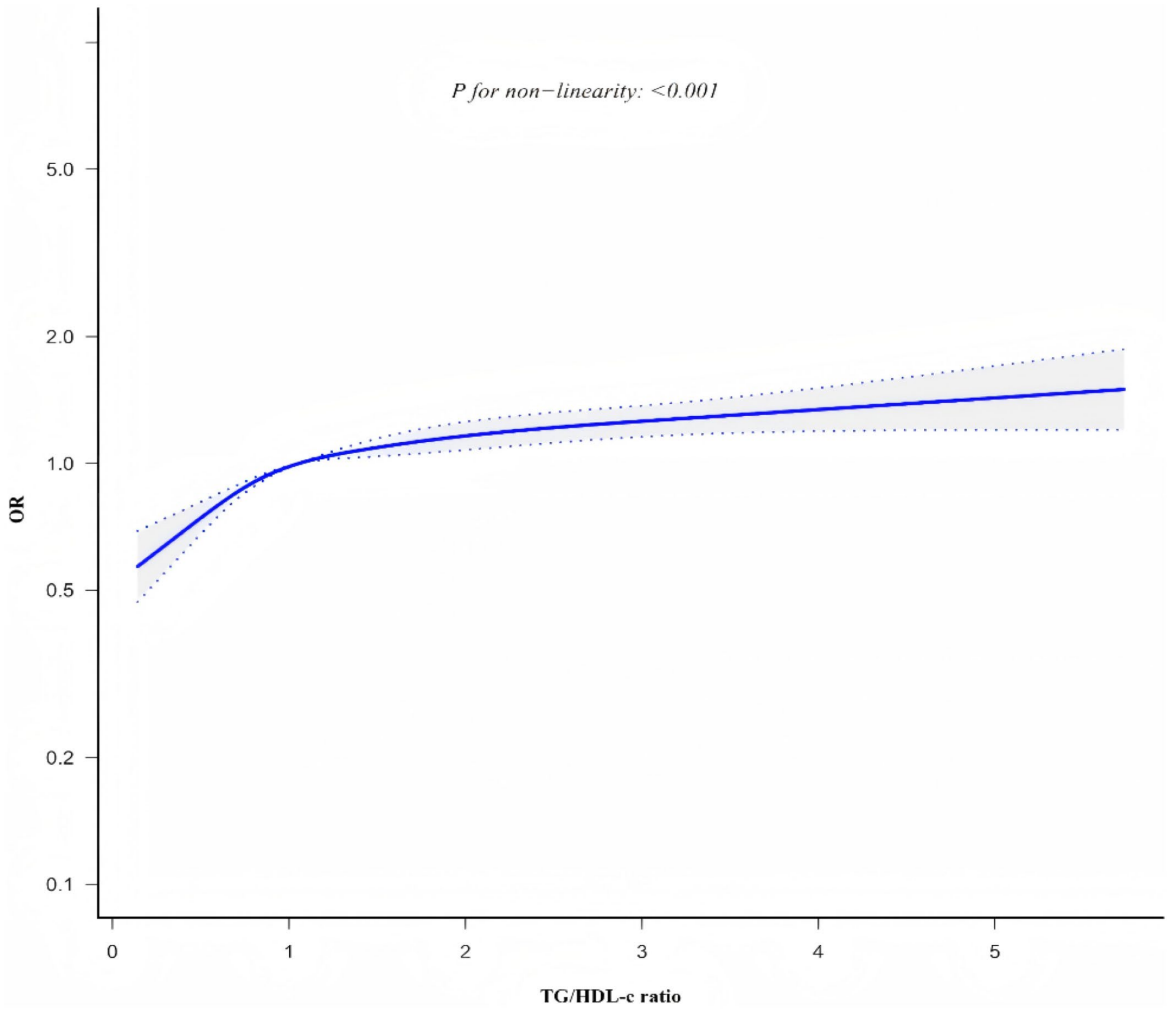
Non-linear relationship between TG/HDL-c ratio and CKD risk.

Generalized additive model and smooth curve fitting revealed a non-linear association between TG/HDL-c ratio and CKD risk (Figure 6). Based on sensitivity analyses using a standard binary logistic regression model, we selected the optimal fit through log-likelihood ratio testing (*p* < 0.05; Table 6). Subsequently, a two-piecewise binary logistic regression model was implemented, with a recursive algorithm identifying an inflection point at 1.086. Analysis revealed distinct associations on either side of this inflection point: below the threshold, the odds ratio was 1.800 (95% CI 1.542 to 2.100), while above it, the association strengthened to 1.094 (95% CI 1.049 to 1.411).

**Table 6:** The result of the two-piecewise linear regression model.

Incident CKD	Model I(OR,95%CI, P)
Fitting model by standard logistic regression	1.186 (1.144, 1.229) < 0.0001
Fitting model by two-piecewise logistic regression	
Inflection point of the TG/HDL-c ratio	1.086
<=Inflection point	1.800 (1.542, 2.100) < 0.0001
> Inflection point	1.094 (1.049, 1.141) < 0.0001
P for log-likelihood ratio test	<0.0001

We adjusted age; sex; BMI; LDL; ALT; AST; WC; GGT; smoking habits, drinking habits; diabetes, hypertension, tumor. OR, odds ratios; CI, confidence, Ref, reference; TG/HDL-c ratio, triglyceride/ high-density lipoprotein cholesterol ratio.

To present the clinical significance of the TG/HDL-c ratio threshold more intuitively, we conducted a group analysis of the participants based on the determined turning point of 1.086 (Supplementary Table S4). The results showed that compared with the group with a TG/HDL-c ratio < 1.086, the metabolic indicators in the group with a TG/HDL-c ratio ≥ 1.086 were significantly worse: the prevalence of hypertension (67.297% vs. 53.383%, *p* < 0.001), diabetes (29.421% vs. 15.903%, *p* < 0.001), and obesity (20.318% vs. 10.170%, *p* < 0.001) was significantly higher; The MAP, BMI, WC, HC, TC, TG, ALT, AST, GGT, Scr, eGFR, as well as male, smoking and drinking proportions were significantly higher (all *p* < 0.001), while HDL-c, UACR and exercise proportions were significantly lower. Particularly importantly, the prevalence of CKD in the group with a TG/HDL-c ratio ≥ 1.086 was significantly higher than that in the group with a ratio < 1.086 (18.951% vs. 13.068%, *p* < 0.001).

Smooth curve plot demonstrating the non-linear association between TG/HDL-c ratio and CKD risk, generated using generalized additive models. The curve shows an inflection point at 1.086, above which the association between TG/HDL-c ratio and CKD risk becomes stronger.

### 3.5 The results of subgroup analyses

To further validate the robustness of our findings presented in Table 2 against potential confounders, we conducted stratified analyses. The results shown in Table 7 demonstrated a highly consistent pattern: the positive association between TG/HDL-c ratio and CKD persisted across all subgroups. Notably, none of the stratified variables exhibited significant effect modification on the relationship between TG/HDL-c ratio and CKD (P-interaction > 0.05 for all stratified variables).

**Table 7:** Effect size of TG/HDL-c ratio on CKD in prespecified and exploratory subgroups.

Characteristic	No of participants	OR (95%CI)	*P* value	P for interaction
Age (years)				0.1732
<40	533	1.179 (0.890, 1.563)	0.2507	
40 to <50	6,776	1.088 (1.001, 1.183)	0.0480	
50 to <60	14,669	1.184 (1.124, 1.247)	<0.0001	
60 to <70	7,858	1.264 (1.160, 1.377)	<0.0001	
≥70	3,462	1.264 (1.160, 1.377)	<0.0001	
Gender				0.5861
Male	10,940	1.189 (1.127, 1.255)	<0.0001	
Female	22,361	1.168 (1.122, 1.216)	<0.0001	
BMI (kg/m^2^)				0.2339
<18.5	788	0.817 (0.536, 1.244)	0.3458	
> = 18.5, <24	14,425	1.157 (1.094, 1.224)	<0.0001	
> = 24, <28	13,051	1.195 (1.139, 1.254)	<0.0001	
> = 28	5,037	1.198 (1.113, 1.289)	<0.0001	
Smoking habits				0.7695
Never smoker	28,094	1.287 (1.245, 1.330)	<0.0001	
Sometimes smoker	853	1.107 (0.933, 1.314)	0.2423	
Regular smoker	4,079	1.263 (1.167, 1.367)	<0.0001	
Not record	275	1.389 (0.954, 2.021)	0.0860	
Drinking habits				0.8685
Never drinker	24,464	1.295 (1.251, 1.340)	<0.0001	
Sometimes drinker	6,291	1.206 (1.120, 1.299)	<0.0001	
Regular drinker	2,284	1.195 (1.071, 1.335)	0.0015	
Not record	262	1.505 (1.077, 2.103)	0.0165	
Tumor				0.6620
Yes	977	1.321 (1.122, 1.555)	0.0008	
No	32,324	1.265 (1.227, 1.303)	<0.0001	
Exercise habits				0.2122
Yes	26,115	1.282 (1.241, 1.325)	<0.0001	
No	7,059	1.191 (1.108, 1.280)	<0.0001	
Not record	127	1.553 (0.999, 2.414)	0.0503	

Above model adjusted forage; sex; BMI; LDL; ALT; AST; WC; GGT; smoking habits, drinking habits; diabetes, hypertension, tumor. In each case, the model is not adjusted for the stratification variable. OR, odds ratios; CI: confidence, Ref: reference; TG/HDL-c ratio triglyceride/ high-density lipoprotein cholesterol ratio.

## 4 Discussion

This study investigated the relationship between TG/HDL-c ratio and CKD in a large, multi-center cohort of the general Chinese population. Our findings demonstrated that elevated TG/HDL-c ratio was independently and positively associated with CKD risk, with this association persisting after adjustment for potential confounders. Importantly, we identified a non-linear relationship with an inflection point at 1.086, where the association was more pronounced below this threshold. These findings suggest that maintaining appropriate TG/HDL-c levels might be a potential target for CKD prevention strategies.

A meta-analysis of observational studies indicates that the global prevalence of CKD is approximately 13.4% (1). In this study, the prevalence of CKD was found to be 15.9%, which is higher than reported levels in the literature. This discrepancy may be attributed to several key factors. Firstly, differences in CKD definition criteria, particularly our inclusion of albuminuria as a diagnostic marker, likely contributed to the higher prevalence rates, as albuminuria is a sensitive indicator of early kidney damage. Secondly, dietary patterns vary significantly between Western and Chinese populations, with Western populations typically consuming more animal protein and high-calorie foods, which may influence the relationship between lipid metabolism and kidney function (46). Additionally, our study population’s demographic characteristics, including a relatively older average age (57.63 years), higher average BMI (24.637 ± 3.692)and a higher proportion of female(67.148%) and patients with hypertension(60.103%), may have influenced the observed prevalence. These population-specific features, combined with variations in study design across different research, could explain the observed differences in CKD prevalence rates (47–50). Therefore, it is acceptable that the prevalence of CKD among the participants in this study is higher than that of the general population.

Our findings demonstrated a significant association between TG/HDL-C ratio and CKD risk, which is largely consistent with previous research while also revealing some novel insights. In a Japanese cross-sectional cohort study of 216,007 adult participants from a national health screening program, the mean TG/HDL-C ratio was 2.66 ± 2.59 in men. Higher TG/HDL-c ratio was associated with a significantly increased risk of CKD (OR = 1.57, 95%CI 1.49–1.65 in men; Female: 1.41, 1.34–1.48, respectively), which was negatively correlated with eGFR and positively correlated with proteinuria. The results remained consistent after adjustment for relevant confounders (23). Another longitudinal cohort study was conducted to investigate the association between TG/HDL-c ratio and renal function decline in 7316 subjects. The results suggested a positive association between the TG/HDL-c ratio and the risk of CKD (OR: 2.56, 95% CI: 1.05–6.38, *p* < 0.05). After adjusting for related confounding factors, it was still found that TG/HDL-c ratio was positively correlated with the risk of renal function decline (OR 1.90, 95%CI 1.21–3.23, *p* = 0.02) (47). Notably, Jia et al. (20) found in a large cross-sectional study higher TG and lower HDL-c were significantly associated with CKD (TG: OR = 1.17, 95%CI: 1.10–1.23; HDL-c: OR = 0.86, 95%CI: 0.79–0.93); Similar association was found between CKD and higher TG/HDL-c ratio (OR = 1.21, 95%CI: 1.13–1.31). Lv et al. (5) found that high TG/HDL-c ratio was an independent risk factor for renal function decline in middle-aged and elderly Chinese patients (OR = 1.90, 95%CI: 1.21–3.23).

However, our study extends beyond previous research by identifying a non-linear relationship between TG/HDL-c ratio and CKD risk, with an inflection point at 1.086. Our findings indicate that when TG/HDL-c < 1.086, for each unit increase in TG/HDL-c, the risk of CKD increases by 80% (OR = 1.800, 95% CI: 1.542 ~ 2.100). Conversely, when TG/HDL-c > 1.086, each unit increase in TG/HDL-c corresponds to a 9% increase in CKD risk (OR = 1.094, 95% CI: 1.049 ~ 1.141). A large number of studies have pointed out that insulin resistance (51), metabolic disease (52), obesity (53), and inflammation (54) are closely related to the risk of CKD, and TG/HDL-c ratio can reflect insulin resistance (55), metabolic disease (56), obesity (57), and inflammation (58). Therefore, high TG/HDL-c level may indicate that patients are in insulin resistance, metabolic disorders, obesity and inflammation, and then increase the risk of CKD. The identification of this non-linear pattern may help explain some of the seemingly conflicting results in the literature (20, 23, 59, 60). In a cross-sectional study involving 2006 participants from the United States (21), a nonlinear relationship between TG/HDL-c ratio and CKD was observed, with an inflection point of 6.68 and odds ratios (95% CI) of 1.08 (1.04 to 1.13) and 0.97 (0.89 to 1.05), respectively. However, other studies have reported no significant association between TG/HDL-c and CKD risk in certain populations (16). These apparent differences in the literature can be attributed to several factors. First, genetic variation between Asian and Western populations may influence patterns of lipid metabolism and response to renal function (61). Second, different dietary habits -Western populations typically consume more animal protein and saturated fat, while Chinese populations follow a more plant-based diet -may affect baseline TG/HDL-C levels and their association with renal function (62). In addition, Asian populations are more sensitive to metabolic disturbances at lower thresholds than Western populations (63), which may explain the earlier onset of the inflection point in our study. Finally, variations in sample size across studies may influence the statistical power to detect complex relationships. Our large-scale study (*n* = 33,301) provided robust statistical power to detect and characterize the non-linear nature of this association, which might have been undetectable in studies with smaller sample sizes.

The elevation of TG/HDL-c ratio is associated with increased CKD risk, though this relationship is likely modulated by participants’ baseline characteristics. As demonstrated in Supplementary Table S4, participants with TG/HDL-c ≥ 1.086 exhibited significantly higher levels of multiple clinical parameters compared to those with TG/HDL-c < 1.086, including age, BMI, liver enzymes (ALT, AST, GGT), lipid profiles (TG, TC), serum creatinine, uric acid, and mean arterial pressure—all established risk factors for CKD (64–66). When TG/HDL-c ratio exceeds 1.086, its relative contribution to CKD risk appears attenuated, potentially due to the concurrent presence of multiple CKD risk factors. Conversely, when TG/HDL-c ratio is below 1.086, the lower levels of other risk factors (including BMI, GGT, TG, AST, ALB, BUN, TC, ALT, and LDL-c) may reduce their confounding effects, thereby amplifying the relative impact of TG/HDL-c ratio on CKD risk.

Recent studies (67) suggest that abnormalities in glucose metabolism and low-grade inflammation significantly modulate the cardiovascular and renal response to lipid derangement independently of LDL-C levels, which are in close agreement with our findings. When the TG/HDL-c ratio exceeded 1.086, the risk of CKD increased by 9% for each unit increase. When the ratio was lower than 1.086, CKD risk increased by 80% per unit increase. This nonlinear relationship may reflect the different mechanisms by which abnormalities in glucose metabolism and low-grade inflammation affect renal function damage at different TG/HDL-c levels. In terms of clinical application, it is recommended that medical staff can use TG/HDL-c ratio ≥1.086 as a screening tool, because only when the TG/HDL-c ratio of patients is controlled below the inflection point, it is possible to reduce the occurrence of CKD by controlling TG/HDL-c ratio. This approach is particularly valuable for resource-limited Settings and primary care Settings, where advanced biomarkers may not be available or expensive. From a public health perspective, the simplicity and cost-effectiveness of calculating the TG/HDL-c ratio make it an ideal tool for mass screening programs aimed at early identification of people at risk of CKD.

Finally, given the bridging role between low-grade inflammation and insulin resistance and CKD, future studies could explore whether anti-inflammatory strategies could reduce the risk of CKD in patients with high TG/HDL-c ratios. This will provide more comprehensive guidance for clinical decision making, focusing not only on lipid regulation but also on interventions targeting inflammation-mediated pathways, especially in patients with preexisting metabolic and inflammatory risks.

Previous studies have shown that an elevated ratio of triglycerides to high-density lipoprotein cholesterol is an indicator of systemic metabolic dysfunction, often accompanied by increased inflammation and enhanced endothelial stress pathways, which are also key factors for cardiovascular and renal damage. We further expanded our findings to reveal that insulin resistance, lipotoxicity, and fat factor signaling pathways may all be involved in the possible mechanism linking triglycerides/high-density lipoprotein cholesterol to chronic kidney disease. The specific mechanism is as follows: (1) Insulin resistance and systemic metabolic dysfunction: This study found that the TG/HDL-c ratio was significantly associated with the risk of CKD (OR = 1.17, 95% CI: 1.13–1.21), which is consistent with existing literature, that is, the TG/HDL-c ratio is a reliable alternative marker for insulin resistance (68). In stratified analysis, we observed that this association was consistent in various subgroups, including different ages, genders, and BMI levels. This indicates that insulin resistance may be a common pathological basis. In particular, when diabetic patients were excluded, the association between TG/HDL-c and CKD remained significant (OR = 1.1848, 95% CI: 1.1230–1.2500), which further supports that TG/HDL-c may affect renal function through mechanisms independent of insulin resistance in diagnosed diabetes. Insulin resistance can damage the kidneys through mechanisms such as high glomerular filtration, podocyte dysfunction, and sodium retention (69); (2) Lipid toxicity and abnormal lipid metabolism: We found that the TG/HDL-c ratio was significantly higher in the CKD group compared to the non-CKD group, suggesting that lipid toxicity may directly contribute to the occurrence of renal injury. When we grouped the participants according to the quartiles of the TG/HDL-c ratio, from the lowest to the highest quartile, the prevalence of CKD showed a significant upward trend (11.1, 14.8, 17.4, and 20.3%, *p* < 0.0001). Abnormal lipid metabolism can lead to abnormal accumulation of lipids in renal cells, causing lipid peroxidation, mitochondrial dysfunction, and endoplasmic reticulum stress (70). At the glomerular level, the accumulation of lipoproteins in the mesangial area can stimulate mesangial cells to produce matrix proteins and pro-inflammatory cytokines, ultimately leading to glomerular sclerosis (71). Our analysis indicates that even in non-obese populations, the association between TG/HDL-c and CKD remains significant (OR = 1.1660, 95% CI: 1.0810–1.2577), which supports that lipid toxicity may function independently of obesity; (3) Endothelial dysfunction and vascular injury: dyslipidemia triggers inflammatory cytokine production and adipokine imbalance (increased leptin, decreased adiponectin), which leads to endothelial dysfunction and vascular injury (72). This endothelial damage affects both kidneys and cardiovascular system simultaneously, with shared pathological processes including oxidative stress, reduced nitric oxide bioavailability, and increased adhesion molecule expression (73). The resulting microvascular dysfunction and tissue fibrosis manifest as progressive renal impairment and cardiovascular complications, establishing TG/HDL-c ratio as a valuable biomarker reflecting systemic vascular health (74); (4) Inflammation and dysregulation of adipokines: Our stratified results showed that compared with TG/HDL-c ratio Q1 group, TG/HDL-c ratio Q4 group had a significantly higher prevalence of obesity (22.08% vs. 7.52%), which reflects the association between adipose tissue dysfunction and the increase in the TG/HDL-c ratio. Adipose tissue dysfunction can lead to imbalance of adipokines, such as increased leptin and decreased adiponectin, triggering a systemic low-grade inflammatory state, which is a key factor in the development of CKD (75, 76).

Inclisiran (77) represents a major advance in lipid-lowering therapy in recent years, and several notable features complement our findings. As a small interfering RNA targeting PCSK9, inclisiran demonstrates potent LDL-C lowering efficacy (up to 50–55% reduction) with the unique advantage of twice-yearly dosing after initial administration. Crucially, unlike some traditional lipid-lowering agents, inclisiran exhibits minimal perturbation of glucose metabolism and inflammatory pathways (78). This favorable profile addresses a critical need for patients with elevated TG/HDL-c ratios, who often exhibit insulin resistance and systemic inflammation—both key mediators in the pathogenesis of CKD.

The potential synergy between our TG/HDL-c threshold and inclisiran therapy is particularly noteworthy in patients with comorbid CKD and glucose metabolism abnormalities. These patients face heightened cardiovascular risk yet are often most vulnerable to adverse effects from conventional therapies that may worsen glycemic control or introduce inflammatory perturbations. For patients with TG/HDL-c ratios exceeding our identified threshold of 1.086, inclisiran may offer a therapeutic option that effectively manages dyslipidemia without exacerbating metabolic or inflammatory dysregulation. We envision a complementary approach in which the TG/HDL-c ratio can be used both as a risk assessment tool for CKD and as a potential biomarker for monitoring treatment response. Clinicians could utilize our threshold of 1.086 to identify high-risk patients, implement targeted interventions such as inclisiran when appropriate, and subsequently monitor changes in the TG/HDL-c ratio as part of treatment assessment. This integration of risk prediction and therapeutic intervention creates a comprehensive framework for managing patients at risk for CKD: 1. Risk Identification: TG/HDL-c ratio ≥1.086 identifies patients at increased CKD risk; 2. Targeted Intervention: Implementation of appropriate therapies, potentially including inclisiran in patients with concomitant elevated LDL-C, particularly those with glucose metabolism abnormalities; 3. Treatment Monitoring: Tracking changes in TG/HDL-c ratio alongside traditional markers of renal function.

Our findings extend the current literature by demonstrating that elevated TG/HDL-c ratio increases CKD risk independently of participants’ normal ranges for age, BMI, LDL-c, HDL-c, and other parameters. Our study’s methodological strength lies in its comprehensive covariate adjustment, which surpasses previous investigations by incorporating lifestyle indicators and additional biochemical markers, including ALT, AST, GGT, and LDL-c—parameters demonstrated to be associated with CKD risk (79, 80). Notably, sensitivity analyses confirmed this association persisted among subjects without pre-existing diabetes, hypertension, or obesity. These robust findings substantiate the independent relationship between TG/HDL-c ratio and CKD risk, providing an evidence-based foundation for clinical interventions targeting TG/HDL-c reduction as a strategy for CKD risk management.

This study exhibits several methodological strengths that enhance the reliability and generalizability of our findings. First, our analysis utilized a large, multi-center cohort (*n* = 33,301) providing robust representation of the Chinese population, ensuring sufficient statistical power and strengthening the external validity of our results. Second, the dataset demonstrates exceptional completeness with minimal missing data across most covariates. Third, our identification of a non-linear relationship between TG/HDL-c ratio and CKD risk, including precise determination of the inflection point, substantially advances current understanding in this field. While the observational nature of our study introduces potential confounding, we implemented rigorous statistical methodology to minimize residual confounding effects. The robustness of our findings was validated through comprehensive sensitivity analyses, including transformation of the primary independent variable, stratified subgroup analyses, incorporation of continuous covariates as smooth curves using GAM, and e-value calculations to quantify potential unmeasured confounding effects.

Our study has several methodological limitations that warrant consideration when interpreting the findings. Our research has some methodological limitations, which need to be taken into account when interpreting the research results. First, according to the KDIGO 2024 guidelines, CKD (24) is defined as abnormalities in kidney structure or function that persist for more than 3 months and have implications for health. We acknowledge that in this study, we were unable to strictly follow the KDIGO guidelines for diagnosing CKD, which is indeed an inherent limitation of cross-sectional studies. However, repeated measurements are often difficult to achieve in large epidemiological surveys, and many well-known cross-sectional studies have faced similar challenges [such as NHANES and China Epidemiological Survey of chronic kidney disease (2)]. To enhance the diagnostic sensitivity, we adopted a dual criterion of eGFR < 60 mL/min/1.73m^2^ and/or UACR ≥ 30 mg/g, which is consistent with recent large-scale epidemiological studies (81, 82). We commit to continuing to conduct GFR, urine protein, urine occult blood, renal structure and 3-month follow-up for patients in future prospective studies, in order to achieve a more accurate and scientific diagnosis of CKD.

Second, during the data collection process, this study failed to record in detail the participants’ use of lipid-lowering drugs, which may affect the interpretation of TG/HDL-c ratio levels, especially for those participants who had received lipid-lowering treatment (30). To address the potential confounding factors brought about by lipid-lowering treatment, we considered that patients with high lipid levels were more likely to receive lipid-lowering treatment. Therefore, we conducted a sensitivity analysis, excluding patients who might be receiving such drug treatment. The results showed that the TG/HDL-c ratio was still significantly associated with CKD (see Supplementary Tables S3, S4). Given the large sample size of this study (*n* = 33,301) and the stability of the results from multiple sensitivity analyses, we believe that this limitation will not fundamentally change our main conclusions. However, for future research, we plan to include drug use data, especially data on lipid-lowering drugs, to more accurately assess the relationship between the TG/HDL-c ratio and the risk of CKD.

Third, despite our efforts to control for established confounders including blood pressure, waist circumference, and body mass index, residual or unmeasured confounding factors may persist—an inherent limitation of observational studies. However, our E-value calculations suggest that the influence of unmeasured confounders would need to be substantial to meaningfully alter our primary conclusions.

Fourth, our study population consisted predominantly of Chinese urban residents who underwent health examinations, which may limit the generalizability of our findings to populations of different ethnicities, socioeconomic backgrounds, or clinical profiles. The applicability of the identified TG/HDL-c threshold (1.086) should be validated in diverse populations before broad implementation.

Fifth, the single measurements of lipid content, urine protein, and glomerular filtration rate in this study may affect the stability of the results, and may underestimate the actual incidence of chronic kidney disease. This is one of the common limitations of cross-sectional studies. However, when the actual incidence of chronic kidney disease is underestimated, multivariate regression analysis shows that the relationship between TG/HDL-c ratio and the risk of CKD remains stable in various sensitivity analyses and stratified analyses. This to some extent supports the reliability of the results of this study. We observed consistent associations in different age, gender, and comorbidity subgroups, which strengthens the robustness of our research results. This method has been recognized in many studies on kidney diseases (17, 28). In future studies, we will repeat the measurement of blood lipid and renal function levels, and dynamically observe the relationship between changes in TG/HDL-c ratio and the risk of CKD.

## 5 Conclusion

In conclusion, this study showed both linear and nonlinear associations of TG/HDL-c with the risk of CKD in a Chinese population. TG/HDL-c ratio >1.086 was independently and positively correlated with CKD. The results of this study may provide clinical guidance for the primary prevention of CKD. TG/HDL-c ratio is a valuable risk assessment measure for CKD, suggesting the potential of threshold-based risk assessment in clinical practice. Future longitudinal studies are needed to confirm these associations and to explore the underlying mechanisms in different populations.

## Data Availability

Publicly available datasets were analyzed in this study. This data can be found here: Data could be downloaded from the PLoS One database (https://journals.plos.org/plosone/).

## Footnotes

^1^ https://journals.plos.org/plosone

## Glossary

CKD: Chronic kidney disease
TG/HDL-c ratio: The ratio of triglycerides to high-density lipoprotein cholesterol
ESRD: End-stage renal disease
CVD: cardiovascular disease
BMI: Body mass index
GAM: Generalized additive model
WC: Waist circumference
HC: hip circumference
Scr: Serum creatinine
TC: Total cholesterol
TG: Triglyceride
ALT: Alanine aminotransferase
AST: Aspartate aminotransferase
HDL-c: High-density lipoprotein cholesterol
LDL-c: Low-density lipoprotein cholesterol
GGT: γ-Glutamyl transpeptidase
FPG: Fasting plasma glucose
PBG: Postprandial blood glucose
SBP: Systolic blood pressure
DBP: Diastolic blood pressure
MAP: Mean arterial blood pressure
DM: Diabetes mellitus
UACR: Urinary albumin-creatinine ratio
eGFR: estimated glomerular filtration rate
MDRD: Modification of Diet in Renal Disease
OR: Odds ratio
Ref: Reference
CI: Confidence intervals
VIF: variance inflation factors

## References

[1] Lv JC Zhang LX. Prevalence and disease burden of chronic kidney disease. Adv Exp Med Biol. (2019) 1165:3–15. doi: 10.1007/978-981-13-8871-2_1 31399958

[2] Zhang L Wang F Wang L Wang W Liu B Liu J. Prevalence of chronic kidney disease in China: a cross-sectional survey. Lancet. (2012) 379:815–22. doi: 10.1016/s0140-6736(12)60033-6 22386035

[3] Slinin Y Greer N Ishani A MacDonald R Olson C Rutks I. Timing of dialysis initiation, duration and frequency of hemodialysis sessions, and membrane flux: a systematic review for a KDOQI clinical practice guideline. Am J Kidney Dis. (2015) 66:823–36. doi: 10.1053/j.ajkd.2014.11.031, PMID: 26498415

[4] Bhalodkar NC Blum S Enas EA. Accuracy of the ratio of triglycerides to high-density lipoprotein cholesterol for predicting low-density lipoprotein cholesterol particle sizes, phenotype B, and particle concentrations among Asian Indians. Am J Cardiol. (2006) 97:1007–9. doi: 10.1016/j.amjcard.2005.10.036 16563906

[5] Lv S Zhang H Chen J Shen Z Zhu C Gu Y. The effect of triglycerides to high-density lipoprotein cholesterol ratio on the reduction of renal function: findings from China health and retirement longitudinal study (CHARLS). Lipids Health Dis. (2021) 20:110. doi: 10.1186/s12944-021-01542-5 34544446 PMC8454112

[6] Ferro CJ Mark PB Kanbay M Sarafidis P Heine GH Rossignol P. Lipid management in patients with chronic kidney disease. Nat Rev Nephrol. (2018) 14:727–49. doi: 10.1038/s41581-018-0072-9, PMID: 30361677

[7] Park B Jung DH Lee HS Lee YJ. Triglyceride to HDL-cholesterol ratio and the incident risk of ischemic heart disease among Koreans without diabetes: a longitudinal study using National Health Insurance Data. Front Cardiovasc Med. (2021) 8:716698. doi: 10.3389/fcvm.2021.716698 34490378 PMC8418107

[8] Zhang X Hu H He L Huang X Zhang Z Tu L. Association between triglyceride to high-density lipoprotein cholesterol ratio and microalbuminuria in the Chinese population. Sci Rep. (2024) 14:30960. doi: 10.1038/s41598-024-82084-5 39730606 PMC11680801

[9] Suh SH Oh TR Choi HS Kim CS Bae EH Oh KH. Serum triglycerides level is independently associated with renal outcomes in patients with non-dialysis chronic kidney disease: results from KNOW-CKD study. Front Nutr. (2022) 9:1037618. doi: 10.3389/fnut.2022.1037618 36505239 PMC9729769

[10] Brunskill NJ. Albumin signals the coming of age of proteinuric nephropathy. J Am Soc Nephrol. (2004) 15:504–5. doi: 10.1097/01.asn.0000112912.40303.81, PMID: 14747400

[11] Vaziri ND. Dyslipidemia of chronic renal failure: the nature, mechanisms, and potential consequences. Am J Physiol Renal Physiol. (2006) 290:F262–72. doi: 10.1152/ajprenal.00099.2005 16403839

[12] Oliveri A Rebernick RJ Kuppa A Pant A Chen Y Du X. Comprehensive genetic study of the insulin resistance marker TG:HDL-C in the UK biobank. Nat Genet. (2024) 56:212–21. doi: 10.1038/s41588-023-01625-2 38200128 PMC10923176

[13] Leyking S Fliser D. Insulin resistance in CKD. Clinic J Am Soc Nephrol. (2014) 9:638–40. doi: 10.2215/cjn.01290214 PMC3974344 24677558

[14] Kim JY Kang HT Lee HR Lee YJ Shim JY. Comparison of lipid-related ratios for prediction of chronic kidney disease stage 3 or more in Korean adults. J Korean Med Sci. (2012) 27:1524–9. doi: 10.3346/jkms.2012.27.12.1524 23255852 PMC3524432

[15] Sandhu S Wiebe N Fried LF Tonelli M. Statins for improving renal outcomes: a meta-analysis. J Am Soc Nephrol. (2006) 17:2006–16. doi: 10.1681/asn.2006010012 16762986

[16] Chawla V Greene T Beck GJ Kusek JW Collins AJ Sarnak MJ. Hyperlipidemia and long-term outcomes in nondiabetic chronic kidney disease. Clin J Am Soc Nephrol. (2010) 5:1582–7. doi: 10.2215/cjn.01450210 20558558 PMC2974397

[17] Nguyen HH Tran HH Nguyen LT Nguyen T Nguyen NA Vi MT. TG/HDL-C ratio is a risk factor associated with CKD: use in assessing the risk of progression of CKD. Pathophysiology. (2022) 29:374–82. doi: 10.3390/pathophysiology29030029, PMID: 35893599 PMC9326757

[18] Kim Y Lee S Lee Y Kang MW Park S Park S. Predictive value of triglyceride/high-density lipoprotein cholesterol for major clinical outcomes in advanced chronic kidney disease: a nationwide population-based study. Clin Kidney J. (2021) 14:1961–8. doi: 10.1093/ckj/sfaa252 34345420 PMC8323149

[19] Weldegiorgis M Woodward M. Elevated triglycerides and reduced high-density lipoprotein cholesterol are independently associated with the onset of advanced chronic kidney disease: a cohort study of 911,360 individuals from the United Kingdom. BMC Nephrol. (2022) 23:312. doi: 10.1186/s12882-022-02932-2 36109725 PMC9479392

[20] Wen J Chen Y Huang Y Lu Y Liu X Zhou H. Association of the TG/HDL-C and non-HDL-C/HDL-C ratios with chronic kidney disease in an adult Chinese population. Kidney Blood Press Res. (2017) 42:1141–54. doi: 10.1159/000485861 29224024

[21] Yu L Zhou L Zhou D Hu G. Nonlinear relationship between triglyceride/high-density lipoprotein cholesterol ratio and chronic kidney disease in US adults: a National Health and nutrition examination survey investigation. Int Urol Nephrol. (2019) 51:2005–14. doi: 10.1007/s11255-019-02287-y 31538278

[22] Kang HT Shim JY Lee YJ Lee JE Linton JA Kim JK. Association between the ratio of triglycerides to high-density lipoprotein cholesterol and chronic kidney disease in Korean adults: the 2005 Korean National Health and nutrition examination survey. Kidney Blood Press Res. (2011) 34:173–9. doi: 10.1159/000323895 21502765

[23] Tsuruya K Yoshida H Nagata M Kitazono T Hirakata H Iseki K. Association of the triglycerides to high-density lipoprotein cholesterol ratio with the risk of chronic kidney disease: analysis in a large Japanese population. Atherosclerosis. (2014) 233:260–7. doi: 10.1016/j.atherosclerosis.2013.12.037, PMID: 24529154

[24] KDIGO. Clinical practice guideline for the evaluation and Management of Chronic Kidney Disease. Kidney Int. (2024) 105:S117–314. doi: 10.1016/j.kint.2023.10.018 38490803

[25] Ye Y Zhang L Yan W Wang A Wang W Gao Z. Self-reported sleep duration and daytime napping are associated with renal hyperfiltration and microalbuminuria in an apparently healthy Chinese population. PLoS One. (2019) 14:e0214776. doi: 10.1371/journal.pone.0214776 31469836 PMC6716775

[26] Chen Z Hu H Chen M Luo X Yao W Liang Q. Association of triglyceride to high-density lipoprotein cholesterol ratio and incident of diabetes mellitus: a secondary retrospective analysis based on a Chinese cohort study. Lipids Health Dis. (2020) 19:33. doi: 10.1186/s12944-020-01213-x 32131838 PMC7057518

[27] Zhou B. Predictive values of body mass index and waist circumference to risk factors of related diseases in Chinese adult population. Zhonghua Liu Xing Bing Xue Za Zhi. (2002) 23:5–10. doi: 10.3760/j.issn:0254-6450.2002.01.003 12015100

[28] American Diabetes Association Professional Practice Committee. 2. Classification and diagnosis of diabetes: standards of medical Care in Diabetes-2020. Diabetes Care. (2020) 43:S14–s31. doi: 10.2337/dc20-S002 31862745

[29] Wang L Xu X Zhang M Hu C Zhang X Li C. Prevalence of chronic kidney disease in China: results from the sixth China chronic disease and risk factor surveillance. JAMA Intern Med. (2023) 183:298–310. doi: 10.1001/jamainternmed.2022.6817 36804760 PMC9941971

[30] Jones GR Imam SK. Validation of the revised MDRD formula and the original Cockcroft and gault formula for estimation of the glomerular filtration rate using Australian data. Pathology. (2009) 41:379–82. doi: 10.1080/00313020902884980, PMID: 19404852

[31] Schaeffner ES Kurth T Curhan GC Glynn RJ Rexrode KM Baigent C. Cholesterol and the risk of renal dysfunction in apparently healthy men. J Am Soc Nephrol. (2003) 14:2084–91. doi: 10.1681/asn.V1482084 12874462

[32] Lee Y Park S Lee S Kim Y Kang MW Cho S. Lipid profiles and risk of major adverse cardiovascular events in CKD and diabetes: a nationwide population-based study. PLoS One. (2020) 15:e0231328. doi: 10.1371/journal.pone.0231328 32271842 PMC7144995

[33] Wax Y. Collinearity diagnosis for a relative risk regression analysis: an application to assessment of diet-cancer relationship in epidemiological studies. Stat Med. (1992) 11:1273–87. doi: 10.1002/sim.4780111003 1518991

[34] Vandenbroucke JP von Elm E Altman DG Gøtzsche PC Mulrow CD Pocock SJ. Strengthening the reporting of observational studies in epidemiology (STROBE): explanation and elaboration. PLoS Med. (2007) 4:e297. doi: 10.1371/journal.pmed.0040297 17941715 PMC2020496

[35] von Elm E Altman DG Egger M Pocock SJ Gøtzsche PC Vandenbroucke JP. The strengthening the reporting of observational studies in epidemiology (STROBE) statement: guidelines for reporting observational studies. Int. J Surg. (2014) 12:1495–9. doi: 10.1016/j.ijsu.2014.07.013 25046131

[36] Rothenbacher D Rehm M Iacoviello L Costanzo S Tunstall-Pedoe H Belch JJF. Contribution of cystatin C- and creatinine-based definitions of chronic kidney disease to cardiovascular risk assessment in 20 population-based and 3 disease cohorts: the BiomarCaRE project. BMC Med. (2020) 18:300. doi: 10.1186/s12916-020-01776-7, PMID: 33161898 PMC7650190

[37] Mullee A Romaguera D Pearson-Stuttard J Viallon V Stepien M Freisling H. Association Between Soft Drink Consumption and Mortality in 10 European Countries. JAMA internal medicine. (2019) 179:1479–90. doi: 10.1001/jamainternmed.2019.2478 31479109 PMC6724165

[38] Keidel D Anto JM Basagaña X Bono R Burte E Carsin AE. The role of socioeconomic status in the Association of Lung Function and air Pollution-a Pooled Analysis of three adult ESCAPE cohorts. Int J Environ Res Public Healthh. (2019) 16:901. doi: 10.3390/ijerph16111901 PMC6603717 31146441

[39] Lee H Kwon SH Jeon JS Noh H Han DC Kim H. Association between blood pressure and the risk of chronic kidney disease in treatment-naïve hypertensive patients. Kidney Res Clinic Pract. (2022) 41:31–42. doi: 10.23876/j.krcp.21.099 PMC8816410 34974658

[40] Shen Y Cai R Sun J Dong X Huang R Tian S. Diabetes mellitus as a risk factor for incident chronic kidney disease and end-stage renal disease in women compared with men: a systematic review and meta-analysis. Endocrine. (2017) 55:66–76. doi: 10.1007/s12020-016-1014-6, PMID: 27477292

[41] Valizadeh M Ahmadi AR Abbaspour F Valizadeh A Syed Hasani AH Moteshakereh SM. The risk of kidney dysfunction in metabolically healthy/unhealthy population with normal weight or overweight/obesity: a systematic review and meta-analysis. Eat Weight Disord. (2024) 29:69. doi: 10.1007/s40519-024-01697-x 39487860 PMC11531429

[42] Zhu F Chen C Zhang Y Chen S Huang X Li J. Elevated blood mercury level has a non-linear association with infertility in U.S. Women: Data from the NHANES 2013–2016. Reproduct Toxicol. (2020) 91:53–8. doi: 10.1016/j.reprotox.2019.11.005 31756438

[43] Haneuse S VanderWeele TJ Arterburn D. Using the E-value to assess the potential effect of unmeasured confounding in observational studies. JAMA. (2019) 321:602–3. doi: 10.1001/jama.2018.21554, PMID: 30676631

[44] Wu L Wu X Hu H Wan Q. Association between triglyceride-to-high-density lipoprotein cholesterol ratio and prediabetes: a cross-sectional study in Chinese non-obese people with a normal range of low-density lipoprotein cholesterol. J Transl Med. (2022) 20:484. doi: 10.1186/s12967-022-03684-1 36273126 PMC9588227

[45] Yang D Lan J Cen J Han Y Hu H. Association between hypertension and new-onset non-alcoholic fatty liver disease in Chinese non-obese people: a longitudinal cohort study. Diabetes Metab Syndr Obesity. (2023) 16:345–63. doi: 10.2147/dmso.S396011 PMC9922508 36788988

[46] Xu SS Hua J Huang YQ Shu L. Association between dietary patterns and chronic kidney disease in a middle-aged Chinese population. Public Health Nutr. (2020) 23:1058–66. doi: 10.1017/s1368980019002805 31576799 PMC7282855

[47] Bikbov B Perico N Remuzzi G. Disparities in chronic kidney disease prevalence among males and females in 195 countries: analysis of the global burden of disease 2016 study. Nephron. (2018) 139:313–8. doi: 10.1159/000489897, PMID: 29791905

[48] Ning Y Shen B Shi Y Song N Fang Y Li Y. Temporal trends in prevalence and mortality for chronic kidney disease in China from 1990 to 2019: an analysis of the global burden of disease study 2019. Clin Kidney J. (2023) 16:312–21. doi: 10.1093/ckj/sfac218 36755850 PMC9900593

[49] Rapp JL Lieberman-Cribbin W Tuminello S Taioli E. Male sex, severe obesity, older age, and chronic kidney disease are associated with COVID-19 severity and mortality in new York City. Chest. (2021) 159:112–5. doi: 10.1016/j.chest.2020.08.2065, PMID: 32866462 PMC7455228

[50] Tao LC Xu JN Wang TT Hua F Li JJ. Triglyceride-glucose index as a marker in cardiovascular diseases: landscape and limitations. Cardiovasc Diabetol. (2022) 21:68. doi: 10.1186/s12933-022-01511-x 35524263 PMC9078015

[51] Spoto B Pisano A Zoccali C. Insulin resistance in chronic kidney disease: a systematic review. Am J Physiol Renal Physiol. (2016) 311:F1087–f1108. doi: 10.1152/ajprenal.00340.2016 27707707

[52] Marassi M Fadini GP. The cardio-renal-metabolic connection: a review of the evidence. Cardiovasc Diabetol. (2023) 22:195. doi: 10.1186/s12933-023-01937-x 37525273 PMC10391899

[53] Jiang Z Wang Y Zhao X Cui H Han M Ren X. Obesity and chronic kidney disease. American journal of physiology. Endocrinol Metab. (2023) 324:E24–e41. doi: 10.1152/ajpendo.00179.2022 36383637

[54] Kadatane SP Satariano M Massey M Mongan K Raina R. The role of inflammation in CKD. Cells. (2023) 12:581. doi: 10.3390/cells12121581 37371050 PMC10296717

[55] Nur Zati Iwani AK Jalaludin MY Yahya A Mansor F Md Zain F Hong JYH. TG: HDL-C Ratio as Insulin Resistance Marker for Metabolic Syndrome in Children With Obesity. Front Endocrinol. (2022) 13:852290. doi: 10.3389/fendo.2022.852290 PMC8965646 35370951

[56] Tian Z Yang L Li Y Huang Y Yang J Xue F. Associations of different insulin resistance-related indices with the incidence and progression trajectory of cardiometabolic multimorbidity: a prospective cohort study from UK biobank. Cardiovascular diabetology. (2025) 24:257. doi: 10.1186/s12933-025-02819-0 40533754 PMC12175334

[57] Widmer A Mercante MG Silver HJ. TG/HDL ratio is an independent predictor for estimating resting energy expenditure in adults with Normal weight, overweight, and obesity. Nutrients. (2022) 14:106. doi: 10.3390/nu14235106 PMC9741411 36501139

[58] Huang Y Zhou Y Xu Y Wang X Zhou Z Wu K. Inflammatory markers link triglyceride-glucose index and obesity indicators with adverse cardiovascular events in patients with hypertension: insights from three cohorts. Cardiovasc Diabetol. (2025) 24:11. doi: 10.1186/s12933-024-02571-x 39780176 PMC11716003

[59] Ho CI Chen JY Chen SY Tsai YW Weng YM Tsao YC. Relationship between TG/HDL-C ratio and metabolic syndrome risk factors with chronic kidney disease in healthy adult population. Clinic Nutr. (2015) 34:874–80. doi: 10.1016/j.clnu.2014.09.007 25304295

[60] Zhang L Yuan Z Chen W Chen S Liu X Liang Y. Serum lipid profiles, lipid ratios and chronic kidney disease in a Chinese population. Int J Environ Res Public Health. (2014) 11:7622–35. doi: 10.3390/ijerph110807622 25075881 PMC4143822

[61] Matey-Hernandez ML Williams FMK Potter T Valdes AM Spector TD Menni C. Genetic and microbiome influence on lipid metabolism and dyslipidemia. Physiol Genomics. (2018) 50:117–26. doi: 10.1152/physiolgenomics.00053.2017 29341867 PMC5867613

[62] Skrypnik K Bogdanski P Kubasik M Wawrzyniak N Markuszewski L Suliburska J. The influence of dietary patterns on arterial stiffness, lipid metabolism, and liver and renal function in the population of greater Poland. Acta Sci Pol Technol Aliment. (2020) 19:301–18. doi: 10.17306/j.Afs.0848 32978913

[63] Ewang-Emukowhate M Perera D Wierzbicki AS. Dyslipidaemia related to insulin resistance and cardiovascular disease in south Asian and west African populations. Curr Pharm Des. (2014) 20:6270–5. doi: 10.2174/1381612820666140620114948 24953401

[64] Ying M Shao X Qin H Yin P Lin Y Wu J. Disease burden and epidemiological trends of chronic kidney disease at the global, regional, National Levels from 1990 to 2019. Nephron. (2024) 148:113–23. doi: 10.1159/000534071, PMID: 37717572 PMC10860888

[65] Chen T Wan H Luo Y Chen L. Association of triglyceride-glucose-body mass index with all-cause and cardiovascular mortality among individuals with chronic kidney disease. Sci Rep. (2024) 14:20593. doi: 10.1038/s41598-024-71579-w 39232126 PMC11375041

[66] Ren X Jiang M Han L Zheng X. Association between triglyceride-glucose index and chronic kidney disease: a cohort study and meta-analysis. Nutr Metabo Cardiovasc Dis. (2023) 33:1121–8. doi: 10.1016/j.numecd.2023.03.026, PMID: 37088649

[67] Di Giacomo Barbagallo F Bosco G Di Marco M Scilletta S Miano N Musmeci M. Evaluation of glycemic status and subclinical atherosclerosis in familial hypercholesterolemia subjects with or without LDL receptor mutation. Cardiovasc Diabetol. (2025) 24:126. doi: 10.1186/s12933-025-02683-y 40114220 PMC11927314

[68] McLaughlin T Reaven G Abbasi F Lamendola C Saad M Waters D. Is there a simple way to identify insulin-resistant individuals at increased risk of cardiovascular disease? Am J Cardiol. (2005) 96:399–404. doi: 10.1016/j.amjcard.2005.03.085 16054467

[69] Artunc F Schleicher E Weigert C Fritsche A Stefan N Häring HU. The impact of insulin resistance on the kidney and vasculature. Nat Rev Nephrol. (2016) 12:721–37. doi: 10.1038/nrneph.2016.145, PMID: 27748389

[70] Ruan XZ Varghese Z Moorhead JF. An update on the lipid nephrotoxicity hypothesis. Nat Rev Nephrol. (2009) 5:713–21. doi: 10.1038/nrneph.2009.184, PMID: 19859071

[71] Muntner P Coresh J Smith JC Eckfeldt J Klag MJ. Plasma lipids and risk of developing renal dysfunction: the atherosclerosis risk in communities study. Kidney Int. (2000) 58:293–301. doi: 10.1046/j.1523-1755.2000.00165.x 10886574

[72] Baragetti A Norata GD Sarcina C Rastelli F Grigore L Garlaschelli K. High density lipoprotein cholesterol levels are an independent predictor of the progression of chronic kidney disease. J Intern Med. (2013) 274:252–62. doi: 10.1111/joim.12081 23607805

[73] Foresto-Neto O Ávila VF Arias SCA Zambom FFF Rempel LCT Faustino VD. NLRP3 inflammasome inhibition ameliorates tubulointerstitial injury in the remnant kidney model. Lab Investig. (2018) 98:773–82. doi: 10.1038/s41374-018-0029-4 29511302

[74] Moradi H Vaziri ND. Molecular mechanisms of disorders of lipid metabolism in chronic kidney disease. Front Biosci. (2018) 23:146–61. doi: 10.2741/4585 28930541

[75] Sharma K. The link between obesity and albuminuria: adiponectin and podocyte dysfunction. Kidney Int. (2009) 76:145–8. doi: 10.1038/ki.2009.137 19404275

[76] Declèves AE Sharma K. Obesity and kidney disease: differential effects of obesity on adipose tissue and kidney inflammation and fibrosis. Curr Opin Nephrol Hypertens. (2015) 24:28–36. doi: 10.1097/mnh.0000000000000087 25470014 PMC4847436

[77] Di Giacomo-Barbagallo F Andreychuk N Scicali R Gonzalez-Lleó A Piro S Masana L. Inclisiran, reasons for a novel agent in a crowded therapeutic field. Curr Atheroscler Rep. (2025) 27:25. doi: 10.1007/s11883-024-01271-x 39786678 PMC11717820

[78] Luo Z Huang Z Sun F Guo F Wang Y Kao S. The clinical effects of inclisiran, a first-in-class LDL-C lowering siRNA therapy, on the LDL-C levels in Chinese patients with hypercholesterolemia. J Clin Lipidol. (2023) 17:392–400. doi: 10.1016/j.jacl.2023.04.010 37164838

[79] Ochiai H Shirasawa T Yoshimoto T Nagahama S Sakamoto K Azuma M. Hepatic steatosis index and chronic kidney disease among middle-aged individuals: a large-scale study in Japan. Dis Markers. (2021) 2021:9941834. doi: 10.1155/2021/9941834, PMID: 34211614 PMC8211514

[80] Lanktree MB Thériault S Walsh M Paré G. HDL cholesterol, LDL cholesterol, and triglycerides as risk factors for CKD: a Mendelian randomization study. Am J Kidney Dis. (2018) 71:166–72. doi: 10.1053/j.ajkd.2017.06.011, PMID: 28754456

[81] Levey AS Stevens LA Schmid CH Zhang YL Castro AF Feldman HI. A new equation to estimate glomerular filtration rate. Ann Internal Med. (2009) 150:604–12. doi: 10.7326/0003-4819-150-9-200905050-00006 19414839 PMC2763564

[82] Webster AC Nagler EV Morton RL Masson P. Chronic kidney disease. Lancet. (2017) 389:1238–52. doi: 10.1016/s0140-6736(16)32064-5 27887750

