# Supplementary material for: Association between the ratio of triglyceride to high-density lipoprotein cholesterol (TG/HDL-c) and chronic kidney disease risk: a large-scale cross-sectional study in a Chinese population
Source: Front Med (Lausanne). 2025 Oct 31;12:1629303. doi: 10.3389/fmed.2025.1629303 (PMC12615472; doi:10.3389/fmed.2025.1629303)
Supplement: Supplementary file 1 [file Table_1.docx]

**Association Between the ratio of triglyceride to high-density lipoprotein cholesterol (TG/HDL-c ) and Chronic Kidney Disease Risk: A Large-Scale Cross-Sectional Study in a Chinese Population**

**Running title: The relationship between TG/HDL ratio and risk of CKD**

**Yu heng Liao^1,2,3^ , Qijun Wan^*1,3^,Hao fei Hu^*1,3^, Haiying Song^*1,2,3^**

^1^ Department of Nephrology, The First Affiliated Hospital of Shenzhen University, Shenzhen 518000, Guangdong Province, China

^2^School of Medicine Shenzhen University, Shenzhen 518000, Guangdong Province, China

^3^Department of Nephrology, Shenzhen Second People’s Hospital, Shenzhen 518000, Guangdong Province, China

***Corresponding author**

**Qijun Wan**

Department of Nephrology,

Shenzhen Second People’s Hospital,

No.3002 Sungang Road, Futian District,

Shenzhen 518000,

Guangdong Province,

China

Tel:+86-755-83366388

E-mail: yiyuan2224@sina.com

***Corresponding author**

**Haofei Hu**

Department of Nephrology,

Shenzhen Second People’s Hospital,

No.3002 Sungang Road, Futian District,

Shenzhen 518000,

Guangdong Province,

China

Tel:+86-755-83366388

E-mail: [huhaofei0319@126.com](mailto:huhaofei0319@126.com)

***Corresponding author**

**Haiying Song**

Department of Nephrology,

Shenzhen Second People’s Hospital,

No.3002 Sungang Road, Futian District,

Shenzhen 518000,

Guangdong Province,

China

Tel:+86-755-83366388

E-mail: [haiyingsong2024@126.com](mailto:haiyingsong2024@162.com)

**Table S1. Collinearity diagnostics steps.**

| **Variable** | VIF  **Step 1** | **Step 2** |
| --- | --- | --- |
|  |  |  |
| **Gender** | 1.8 | 1.7 |
| **Age(years)** | 1.5 | 1.5 |
| **BMI** | 3 | 3 |
| **MAP** | 2.1 | 2.1 |
| **Smoking habits** | 1.5 | 1.5 |
| **Drinking habits** | 1.4 | 1.4 |
| **Working habits** | 1.3 | 1.3 |
| **ALT(U/L)** | 3.2 | 3.2 |
| **AST(U/L)** | 3.2 | 3.1 |
| **WC (cm)** | 3 | 3 |
| **GGT (U/L)** | 1.3 | 1.3 |
| **TC(mmol/L)** | 6.2 | NA |
| **LDL-c(mmol/L)** | 6.1 | 1.1 |
| **Diabetes** | 1.1 | 1.1 |
| **Hypertension** | 2.2 | 2.2 |
| **Obesity** | 1.9 | 1.9 |
| **Tumor** | 1 | 1 |
|  | 1.0 | 1.0 |

TG/HDL-c ratio triglyceride/ high-density lipoprotein cholesterol ratio;BMI body mass index, MAP mean arterial blood pressure; WC waist circumference ; TC total cholesterol;ALT alanine aminotrans ferase, AST aspartate aminotrans ferase;GGT γ-glutamyl transpeptidase;

Abbreviation: VIF: variance inflation factor; VIF = 1/(1-R^2^).

Note: The variables with VIF>5 will be regarded as collinear variables and cannot be included in the multiple regression model.

**Table S2 .Relationship between TG/HDL-c ratio and CKD under different models in the population with TG < 1.7 mmol/L**

| Variable | Model I (OR,95%CI, P) | Model II(OR,95%CI, P) | Model III (OR,95%CI, P) |
| --- | --- | --- | --- |
| **TG/HDL-c** | 1.635 (1.483, 1.802) <0.00001 | 1.577 (1.421, 1.751) <0.00001 | 1.441 (1.294, 1.604) <0.00001 |
| **TG/HDL-c**  **(Quintile)** |  |  |  |
| Q1 | Ref | Ref | Ref |
| Q2 | 1.370 (1.250, 1.502) <0.00001 | 1.287 (1.171, 1.413) <0.00001 | 1.302 (1.183, 1.433) <0.00001 |
| Q3 | 1.599 (1.446, 1.767) <0.00001 | 1.497 (1.348, 1.662) <0.00001 | 1.437 (1.291, 1.600) <0.00001 |
| Q4 | 1.576 (1.272, 1.954) 0.00003 | 1.578 (1.264, 1.969) 0.00005 | 1.330 (1.059, 1.670) 0.01424 |
| **P for trend** | <0.00001 | <0.00001 | <0.00001 |

Model I: we did not adjust other covariates;

Model II: we adjust age; sex; BMI;

Model III: we adjust age; sex; BMI; LDL; ALT; AST; WC; GGT; smoking habits, drinking habits; diabetes, hypertension, tumor;

**Table S3 .Relationship between TG/HDL-c ratio and CKD under different models in the population with LDL-c < 3.12mmol/L**

| Variable | Model I (OR,95%CI, P) | Model II(OR,95%CI, P) | Model III (OR,95%CI, P) |
| --- | --- | --- | --- |
| **TG/HDL-c** | 1.190 (1.149, 1.233) <0.00001 | 1.174 (1.131, 1.219) <0.00001 | 1.115 (1.072, 1.160) <0.00001 |
| **TG/HDL-c**  **(Quintile)** |  |  |  |
| Q1 | Ref | Ref | Ref |
| Q2 | 1.380 (1.230, 1.549) <0.00001 | 1.301 (1.157, 1.464) 0.00001 | 1.262 (1.120, 1.422) 0.00013 |
| Q3 | 1.565 (1.396, 1.756) <0.00001 | 1.441 (1.279, 1.623) <0.00001 | 1.340 (1.186, 1.513) <0.00001 |
| Q4 | 1.760 (1.581, 1.959) <0.00001 | 1.626 (1.452, 1.821) <0.00001 | 1.428 (1.270, 1.607) <0.00001 |
| **P for trend** | <0.00001 | <0.00001 | <0.00001 |

Model I: we did not adjust other covariates;

Model II: we adjust age; sex; BMI;

Model III: we adjust age; sex; BMI; LDL; ALT; AST; WC; GGT; smoking habits, drinking habits; diabetes, hypertension, tumor;

**Table S4. The characteristics of participants on both sides of the inflection point.**

|  | **TG/HDL-c low level <1.086** | **TG/HDL-c High level >=1.086** | **P-value** |
| --- | --- | --- | --- |
| **Participants** | 17217 | 16084 |  |
| **Age (years)** | 57.0 ± 9.3 | 58.4 ± 9.2 | <0.001 |
| **Gender, n(%)** |  |  | <0.001 |
| Male | 4863 (28.245) | 6077 (37.783) |  |
| Female | 12354 (71.755) | 10007 (62.217) |  |
| **BMI (kg/m^2^)** | 23.8 ± 3.6 | 25.6 ± 3.6 | <0.001 |
| **MAP (mmHg)** | 93.8 ± 12.7 | 97.6 ± 12.5 | <0.001 |
| **WC (cm)** | 83.6 ± 9.9 | 88.8 ± 9.4 | <0.001 |
| **HC (cm)** | 95.5 ± 7.7 | 98.6 ± 7.6 | <0.001 |
| **HDL-c (mmol/L)** | 1.5 ± 0.3 | 1.1 ± 0.2 | <0.001 |
| **LDL-c (mmol/L)** | 3.0 ± 0.9 | 3.0 ± 0.9 | 0.218 |
| **TC (mmol/L)** | 5.0 ± 1.1 | 5.1 ± 1.2 | <0.001 |
| **TG(mmol/L)** | 1.0 ± 0.3 | 2.2 ± 0.9 | <0.001 |
| **ALT (U/L)** | 0.7 ± 0.2 | 2.0 ± 0.9 | <0.001 |
| **AST (U/L)** | 14.0 (10.0-19.0) | 16.0 (12.0-23.0) | <0.001 |
| **GGT (U/L)** | 18.0 (14.0-26.0) | 24.0 (17.0-37.0) | <0.001 |
| **Scr (mmol/L)** | 64.4 (58.6-71.7) | 67.2 (60.4-76.2) | <0.001 |
| **eGFR (ml/min/1.73m^2^)** | 66.7 ± 15.2 | 70.2 ± 18.3 | <0.001 |
| **UACR (mg/g)** | 95.2 ± 18.2 | 92.8 ± 19.7 | 0.006 |
| **Smoking habits, n(%)** |  |  | <0.001 |
| Never smoker | 14983 (87.024) | 13111 (81.516) |  |
| Sometimes smoker | 368 (2.137) | 485 (3.015) |  |
| Regular smoker | 1736 (10.083) | 2343 (14.567) |  |
| Not record | 130 (0.755) | 145 (0.902) |  |
| **Drinking habits, n(%)** |  |  | 0.017 |
| Never drinker | 12776 (74.20) | 11688 (72.668) |  |
| Sometimes drinker | 3159 (18.348) | 3132 (19.473) |  |
| Regular drinker | 1147 (6.662) | 1137 (7.069) |  |
| Not record | 135 (0.784) | 127 (0.790) |  |
| **Exercise habits, n(%)** |  |  | <0.001 |
| Yes | 3847 (22.344) | 3212 (19.970) |  |
| No | 13301 (77.255) | 12814 (79.669) |  |
| Not record | 69 (0.401) | 58 (0.361) |  |
| **Tumor, n(%)** | 502 (2.9) | 475 (3.0) | 0.839 |
| **CKD, n(%)** | 2250 (13.068) | 3048 (18.951) | <0.001 |
| **hypertension, n(%)** | 9191 (53.383) | 10824 (67.297) | <0.001 |
| **Diabetes, n(%)** | 2738 (15.903) | 4732 (29.421) | <0.001 |
| **Obesity, n(%)** | 1751 (10.170) | 3268 (20.318) | <0.001 |

Values are n (%) or mean ± SD or median (quartile)；

BMI body mass index, MAP mean arterial blood pressure; WC waist circumference ; HC hip circumference;

TC total cholesterol,  TG triglyceride, HDL-c high-density lipoprotein cholesterol, LDL-c low-density lipoprotein cholesterol, TG/HDL-c ratio triglyceride/ high-density lipoprotein cholesterol ratio, ALT alanine aminotransferase, AST aspartate aminotransferase, Scr serum creatinine, GGT γ-glutamyl transpeptidase; eGFR Glomerular Filtration Rate; UACR Random urinary protein/creatinine ratio; OR  Odds ratios, CI confidence interval, Ref reference;
